# Regulation of PD-L1 Expression by YY1 in Cancer: Therapeutic Efficacy of Targeting YY1

**DOI:** 10.3390/cancers16061237

**Published:** 2024-03-21

**Authors:** Ana Dillen, Indy Bui, Megan Jung, Stephanie Agioti, Apostolos Zaravinos, Benjamin Bonavida

**Affiliations:** 1 Department of Microbiology, Immunology & Molecular Genetics, Jonsson Comprehensive Cancer, David Geffen School of Medicine, University of California, Los Angeles, CA 90095, USA; anajae@g.ucla.edu (A.D.); indybui500@gmail.com (I.B.);; 2 Cancer Genetics, Genomic and Systems Biology Group, Basic and Translational Cancer Research Center (BTCRC), 1516 Nicosia, Cyprusa.zaravinos@euc.ac.cy (A.Z.); 3 Department of Life Sciences, School of Sciences, European University Cyprus, 2404 Nicosia, Cyprus

**Keywords:** cancer, resistance, YY1, PD-L1, immunotherapy, T cells, inhibitors, overexpression

## Abstract

**Simple Summary:**

We have recently witnessed several milestones in the treatment of various cancers with immunotherapy through modulating the activity of the immune system to suppress and destroy cancer cells. One main factor involved in the anti-cancer immune system is cytotoxic immune cells known as T cells. T cells can recognize pathogenic cancer cells and launch targeted destruction attacks. However, these T cells may become non-functional due to their close association with cancer cells within the tumor microenvironment. Cancer cell activity can ultimately suppress T-cell function. New approaches that prevent the inactivation of T cells by cancer cells would result in recovery of the T-cell functions, cancer regression, and overall survival. Targeting cancer cells specifically to prevent T-cell inactivation would result in tumor disappearance, inhibition of metastasis, and the reversal of resistance to therapy. Therefore, this proposed alternative approach is promising as it is applicable to numerous cancers, including those that are resistant to treatment.

**Abstract:**

During the last decade, we have witnessed several milestones in the treatment of various resistant cancers including immunotherapeutic strategies that have proven to be superior to conventional treatment options, such as chemotherapy and radiation. This approach utilizes the host’s immune response, which is triggered by cancer cells expressing tumor-associated antigens or neoantigens. The responsive immune cytotoxic CD8^+^ T cells specifically target and kill tumor cells, leading to tumor regression and prolongation of survival in some cancers; however, some cancers may exhibit resistance due to the inactivation of anti-tumor CD8^+^ T cells. One mechanism by which the anti-tumor CD8^+^ T cells become dysfunctional is through the activation of the inhibitory receptor programmed death-1 (PD-1) by the corresponding tumor cells (or other cells in the tumor microenvironment (TME)) that express the programmed death ligand-1 (PD-L1). Hence, blocking the PD-1/PD-L1 interaction via specific monoclonal antibodies (mAbs) restores the CD8^+^ T cells’ functions, leading to tumor regression. Accordingly, the Food and Drug Administration (FDA) has approved several checkpoint antibodies which act as immune checkpoint inhibitors. Their clinical use in various resistant cancers, such as metastatic melanoma and non-small-cell lung cancer (NSCLC), has shown significant clinical responses. We have investigated an alternative approach to prevent the expression of PD-L1 on tumor cells, through targeting the oncogenic transcription factor Yin Yang 1 (YY1), a known factor overexpressed in many cancers. We report the regulation of PD-L1 by YY1 at the transcriptional, post-transcriptional, and post-translational levels, resulting in the restoration of CD8^+^ T cells’ anti-tumor functions. We have performed bioinformatic analyses to further explore the relationship between both YY1 and PD-L1 in cancer and to corroborate these findings. In addition to its regulation of PD-L1, YY1 has several other anti-cancer activities, such as the regulation of proliferation and cell viability, invasion, epithelial–mesenchymal transition (EMT), metastasis, and chemo-immuno-resistance. Thus, targeting YY1 will have a multitude of anti-tumor activities resulting in a significant obliteration of cancer oncogenic activities. Various strategies are proposed to selectively target YY1 in human cancers and present a promising novel therapeutic approach for treating unresponsive cancer phenotypes. These findings underscore the distinct regulatory roles of YY1 and PD-L1 (*CD274*) in cancer progression and therapeutic response.

## 1. Introduction

As a major and complex disease, cancer consists of over a hundred different subtypes with various phenotypes, etiologies, and treatment strategies. The current common treatments for cancer consist of surgery, radiation, hormonal therapy, chemotherapy, and immunotherapy. Though these various conventional treatment strategies have resulted in significant clinical responses in a large subset of patients, cancer drug resistance remains a substantial complication. A significant subset of cancer patients is either initially unresponsive to therapies or acquires treatment resistance following initial therapy exposures [1,2]. Thus, unresponsive patients require novel therapies to overcome therapeutic resistance and suppress cancer activity. The evolution of these therapies must also align with the concurrent advancement of innovative targeting strategies. Such therapies should be designed to specifically target underlying factors accountable for therapy resistance and include small-molecule inhibitors/activators [3,4] and anti-tumor monoclonal antibodies (mAbs) [5] that activate the host anti-tumor immune response [6,7,8].

In many therapy-resistant cancer phenotypes, the anti-cancer immune response is suppressed or inactivated [9,10]. Mechanisms that are tied to cancer immune unresponsiveness and evasion involve the immunosuppressive environment of the tumor microenvironment (TME) [6,11,12]. The TME holds several immunosuppressive cells and factors including cancer-associated fibroblasts (CAFs) [13,14], myeloid-derived suppressor cells (MDSCs) [15,16], tumor-associated macrophages (TAMs) [17,18], T regulatory cells (Tregs) [19,20], and other additional suppressive factors such as transforming growth factor-β (TGF-β) [21,22], chemokines, cytokines [23,24], etc. 

Anti-tumor CD8^+^ T cells express an inhibitory receptor, programmed cell death receptor 1 (PD-1). PD-1, a receptor found on immune T cells, B cells, and macrophages, plays a role in regulating the immune system to maintain immune tolerance through signaling and also prevents the negative effects of prolonged immune responses [25,26,27,28]. PD-1 on T cells interacts with programmed cell death ligands 1 (PD-L1) or 2 (PD-L2). PD-L1 and PD-L2 are typically expressed by several other immune cells during inflammatory responses; however, these ligands are also often expressed by cancer cells or tumor-associated cells. Activation of PD-1 by PD-L1 or PD-L2 results in the inactivation of the cytotoxic functions of anti-tumor CD8^+^ T cells, leading to tumor progression and cancer cell invasion [29,30]. Therefore, blocking the interaction between PD-1 and PD-L1/2 can restore the anti-tumor immune activity and allow for tumor regression [31,32]. Many pre-clinical and clinical findings have allowed for the development of Food and Drug Administration (FDA)-approved checkpoint inhibitor mAbs directed against PD-1 and PD-L1. This novel therapeutic approach has been beneficial in targeting chemoresistant cancers, including melanoma, lung cancers, and several other human cancers [31,33].

Another approach to blocking the PD-1/PD-L1/2 CD8^+^ T-cell inactivation focuses on inhibiting the induction of PD-1 on T cells or inhibiting the expression of PD-L1/2 in tumor cells. Of importance, the expression of PD-L1 on tumor cells has been demonstrated to be regulated by the overexpression of the transcription factor (TF) Yin Yang 1 (YY1) [34,35,36,37,38,39]. YY1 is known to be implicated in the pathogenesis of various cancer cells as an immunosuppressive factor [40]. In this report, we address the underlying mechanisms of YY1 in the regulation of PD-L1 expression on cancer cells and determine whether its inhibition will restore not only the anti-tumor activities of CD8^+^ T cells but also reverse other pro-tumorigenic activities associated with YY1, such as its role in tumor cell proliferation, cell viability, tumor invasion, epithelial–mesenchymal transition (EMT), metastasis, and chemo-immuno-resistance.

## 2. YY1 Overexpression in Cancer, Oncogenic and Immunosuppressive Properties

**Figure 1:**
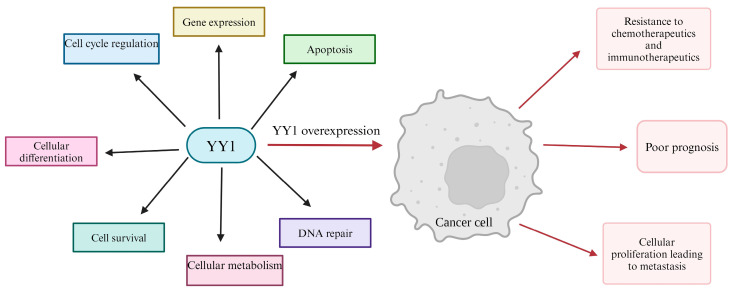
YY1 regulation of cellular processes and its role in cancer cells. YY1 is an activator or repressor in several cellular processes including gene expression, apoptosis, DNA repair, cellular metabolism, cell survival, cellular differentiation, and cell cycle regulation. Disruption of YY1 signaling and overexpression of YY1 in cancer is associated with metastasis, resistance to chemotherapy and immunotherapy in cancer patients, and poor prognosis in patients [55,56]. Figure 1, Figure 2, Figure 3, Figure 4, Figure 5 and Figure 6 were generated using https://www.biorender.com/ accessed on 12 January 2023.

The transcription factor YY1 plays a critical role in regulating a number of cellular processes [41,42,43,44]. The name “Yin Yang” is due to its dual context-dependent abilities in both transcriptional activation and repression. YY1 belongs to the zinc finger transcription factor in the GLI-Kruppel class and is commonly found in enhancer and promoter regions of DNA [41,45,46,47]. The *YY1* gene is 23 kb long and is highly conserved across cells; its translated protein consists of 414 amino acids [45,46,47]. Due to its roles in the cell cycle, gene expression, DNA repair, cellular metabolism, differentiation, survival, and apoptosis, YY1 is vital in early developmental processes as well as lifelong regulatory processes [48,49,50]. However, it has also been found that YY1 is overexpressed in many types of cancers, specifically in several metastatic cancers [51,52,53,54]. This overexpression of YY1 is associated with poor prognosis and resistance to both chemo- and immunotherapeutics. Though YY1 has been determined to be linked to cancer progression and treatment resistance, the exact downstream effects of YY1 overexpression in cancer are still being explored [55,56] (See Figure 1).

YY1, a ubiquitously expressed transcription factor, plays a crucial role in proliferation, cell survival, development, and differentiation through its interaction with several complexes [46,56,57]. The cell cycle is regulated by YY1 through its promotion or suppression of a number of genes [58]. Additionally, YY1 promotes cell survival by regulating anti-apoptotic factors and signaling pathways [59,60,61]. Cell development and differentiation are also influenced by YY1 due to its control of gene expression patterns [41,44,62,63]. Therefore, the overexpression of YY1 results in dysfunction of these regulatory loops, leading to poor health outcomes [56,64,65]. One relevant pathway is the nuclear factor kappa B (NF-κB)/Snail/YY1/Raf kinase inhibitory protein (RKIP) loop, which regulates the EMT [51,52,53,54]. NF-κB stimulates overexpression of YY1, leading to transcriptional activation of Snail and activation of downstream EMT-related molecules [51,66,67,68,69,70]. EMT dysregulation contributes to cancer metastasis through the disruption of cell–cell adhesion and the promotion of a migratory cellular state [51,66,71]. Another factor implicated with YY1 is the TF specificity protein 1 (Sp1), which acts as a coactivator to form a YY1-Sp1 complex [56,72]. This initiates the transcription of the mu opioid receptor (*MOR*) gene in human lymphocytes [56,72]. The activation of *MOR* causes the suppression of lymphocyte proliferation. Therefore, overexpression of YY1 may contribute to the suppression of lymphocyte proliferation through the upregulation of *MOR* expression [56,73].

Although YY1 may act as an activator, it also has many important inhibitory roles, one of which involves the protein p53 which is critical to the regulation of the cell cycle. YY1 can directly bind to the ACAT sequence of p53 and inhibit its function by displacing transcriptional activators [74]. The protein p53 is a checkpoint for cellular division and triggers autophagy in damaged cells [48]. Further, the loss of p53 function occurs in more than 50% of tumors and is a primary indicator of failure of clinical responses [75,76]. Overexpression of YY1 may cause dysfunction within the cell cycle if p53 is overly inhibited. Additionally, the overexpression of YY1 leads to the stimulation of p53 ubiquitination and degradation [74,77]. This inhibition is achieved via disruption of p53 and its coactivator p300 [77]. However, the interaction between p300 and YY1 is context-dependent [57,78,79]. Acetylation of YY1 through p300 results in transcriptional activation, while deacetylation through histone deacetylases (HDACs) results in transcriptional repression [57,78,79]. Additionally, YY1 has been found to directly bind to the p53-half-binding sites in the *p21* and *GADD45* genes, which regulate growth arrest and DNA repair [80]. This regulatory relationship between YY1 and p53 is important in understanding the oncogenic potential of YY1 in human cancers. 

Furthermore, YY1 can inhibit apoptosis through several mechanisms, contributing to cancer cell survival and chemoresistance. One potential molecule that has been shown to be negatively regulated by YY1 is Fas, an inducer of apoptosis [81]. Transcriptional repression of the *Fas* gene causes resistance to Fas-induced apoptosis by cytotoxic CD8^+^ T cells [81,82]. A study conducted by Pothoulakis and colleagues [82] on colorectal cancer (CRC) patients revealed that upregulation of YY1 resulted in the repression of host immunosurveillance mechanisms through Fas downregulation on tumor cells, and that mRNA expression levels of YY1 and Fas were inversely correlated. Additionally, it was found that YY1 is negatively regulated by corticotropin-releasing hormone receptor-2 (CRHR2)/Urocortin-2 (Ucn2) signaling, which can result in transcriptional de-repression of Fas and can resensitize the tumor to anti-Fas antibody-mediated apoptosis [82]. CRHR2/Ucn2 was identified as a regulator of Fas transcriptional de-repression via microRNA (miRNA) mediated inhibition of YY1 [82]. Galloway et al. [83] reported that YY1 regulates the expression of survivin. YY1 also negatively regulates the transcription of death receptor 5 (DR5) in CD8^+^ T cells. YY1 suppression of DR5 can lead to resistance to tumor necrosis factor-related apoptosis-inducing ligand (TRAIL)-induced apoptosis [64]. YY1 interacts with the binding site on the DR5 promoter by recruiting histone-acetyl-transferase, histone-deacetylase, and histone-methyl-transferase enzymes, and may direct histone acetylation, deacetylation, and methylation [67,84]. Furthermore, upregulation of DR5 and resensitization to TRAIL-mediated apoptosis were observed following YY1 inhibition [67]. The consequences of YY1 overexpression in various cancers are still being explored and its many oncogenic and immune-suppressive properties make it a promising potential therapeutic target.

## 3. Role of the PD-1/PD-L1 Interaction in the Inactivation of Anti-Tumor CD8^+^ T Cells and Immune Escape

Cancer immune evasion presents a significant challenge in immunotherapy; thus, understanding these mechanisms of immune escape is vital in creating successful outcomes from treatment [85,86]. Additionally, the T-cell-mediated immune response plays an important role in fighting the body’s infected or cancerous cells [87]. Recent advances in T-cell therapies have focused on extracting and amplifying the number of T cells that recognize tumor cells in order to enhance the immune response [87,88,89]. Certain receptors on T cells act as immune checkpoints, regulating activity levels and limiting tissue damage [90,91]. One important regulatory immune checkpoint is PD-1. Stimulation of PD-1 by PD-L1 or PD-L2 induces inhibition of T-cell proliferation and T-cell activity [87,91]. Consequently, overexpression of PD-L1 in tumor cells can lead to PD-1 activation and suppression of T-cell immune response within the TME [87,91].

PD-1 is a transmembrane protein consisting of 288 amino acids and an extracellular N-terminal domain [29,92,93]. As a receptor on several immune cells, it serves as an inhibitor of both adaptive and innate immune responses [29,92]. It is prominently expressed on T cells but can also be found on natural killer (NK) cells, B lymphocytes, macrophages, dendritic cells (DCs), and monocytes [29,92]. Its ligand PD-L1 is a transmembrane glycoprotein made up of 290 amino acids that is commonly expressed by macrophages, some activated T and B cells, DCs, and some epithelial cells [29,94]. The expression of PD-1 is a marker of activated T cells and plays an important role in reducing the regulation of ineffective or harmful immune responses. However, in the context of cancer immune evasion [29,95], its expression by tumor cells can lead to the development of an adaptive immune mechanism that evades anti-tumor responses [29,96,97].

**Figure 2:**
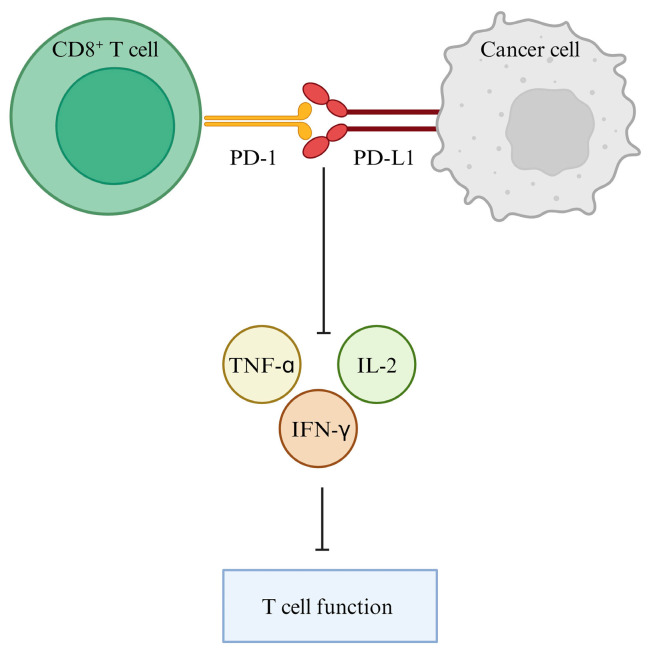
Effect of the PD-L1/PD-1 interaction on T-cell immune function and cytokines. The modulation of the immune T-cell system occurs when the immune checkpoint PD-L1, present on tumor cells, interacts with PD-1 on CD8 T cells. The interaction of PD-1 and PD-L1 blocks and suppresses T-cell function, including anti-tumor CD8 T cells. Additionally, PD-L1/PD-1 leads to a decrease in the cytokines TNF-α, IFN-γ, and IL-2, allowing for immune escape in tumor cells.

T-cell exhaustion can also contribute to the progression of tumorigenesis and failure of long-term responses to immunotherapy [98,99,100]. The effects of T-cell exhaustion manifest in decreased proliferation, increased expression of inhibitory checkpoint molecules such as PD-1, impaired production of type I cytokines, and reduced ability to kill antigenic targets [101,102,103]. The inactivation of PD-1-expressing CD8^+^ T cells by PD-L1 inhibits tumor-infiltrating CD4^+^/CD8^+^ T cells (CD4^+^/CD8^+^ TILs) and leads to a decrease in cytokines including tumor necrosis factor-alpha (TNF-α), interferon gamma (IFN-γ), and interleukin 2 (IL-2), allowing cancer cells to escape the immune reaction [104,105,106,107]. In a study by Baitsch et al. [101], melanoma samples were found to have a majority of their tumor-infiltrating lymphocytes (TILs) exhausted from PD-1 compared to normal T-cell function in non-cancerous skin cells [101]. It was concluded that upregulation of PD-L1 expression is a mechanism for tumor cells to defend themselves from cell death by cytotoxic T cells [104,105,106,107] (Figure 2).

Given the PD-1/PD-L1 axis’ association with immune activity suppression and its downstream pro-cancer effects, both PD-1 and PD-L1 have been the focus of several targeted therapies. Checkpoint proteins such as PD-1 and PD-L1 act to dampen the immune system, blocking harmful immune responses. In cancer, overexpressed PD-L1 by tumor cells hijacks this immune regulatory system, preventing proper targeting and response against cancer cells. Therefore, by blocking these checkpoint proteins, the immune system can recognize and target cancer cells more effectively [66,108,109,110]. PD-1/PD-L1-targeted therapy is an approved and successful checkpoint inhibitor therapy used to treat cancer [111,112,113,114]. Zhou et al. [115] explored this implication in castration-resistant prostate cancer (CRPC) by finding a positive correlation between the expression of PD-L2 and the protein heterogeneous nuclear ribonucleoprotein L (HnRNP L). Thus, decreasing HnRNP L levels reduces PD-L1 expression in cancer cells, making them more vulnerable to T-cell attacks. As a potential therapeutic agent, HnRNP L knockdown as a PD-1/PD-L1 blockade strategy may enhance immune checkpoint inhibitor treatment for CRPC [115]. 

Several immune checkpoint inhibitors, such as those targeting cytotoxic T-lymphocyte-associated protein-4 (CTLA-4), PD-1, and PD-L1, have demonstrated significant promise in improving immunotherapies [8,31,116,117,118,119]. Ipilimumab is one of the drugs that target the immune checkpoint CTLA-4, while Nivolumab targets PD-1; in some trials, various immune checkpoint inhibitors are used in combination with one another or with other therapeutics [111,120,121]. Combination therapies such as Ipilimumab + Nivolumab have been demonstrated to be more effective than single-checkpoint inhibitor monotherapies alone [122,123]. Atezolizumab, Avelumab, and Durvalumab, on the other hand, are designed to solely target PD-L1 [124]. Inhibitors targeting PD-L1 or PD-1 have proven effective in blocking the PD-1/PD-L1 axis, leading to improved response rates in numerous cancer patients [108,125,126]. Despite success in certain cancers, this approach poses challenges, as immune checkpoint inhibitors targeting tumor-specific CD8^+^ T cells may inadvertently impact non-tumor-specific immune responses. This modulation of the immune system may risk autoimmunity and harm to healthy tissues [127,128,129]. Notably, immune-related adverse events most frequently manifest as dermatological conditions or gastrointestinal distress, with common outcomes including hepatotoxicity, endocrinopathies, pneumonitis, pancreatitis, and neurotoxicity [130,131]. Therefore, due to the toxicities associated with checkpoint inhibitors, it is important to consider specificity in immune checkpoint treatments.

## 4. Regulation of PD-L1 by YY1

### 4.1. Transcriptional

In a study by Zhang et al., YY1 was observed as a transcriptional regulator of PD-L1 in human trophoblasts [132]. They found that YY1 can directly bind to the *CD274* gene and associate specifically with the promoter region. Additionally, it was also shown in vivo that decreased YY1 levels in trophoblasts resulted in lower PD-L1 levels [132]. YY1 may also indirectly regulate PD-L1 through control over several other factors. Specifically, the intracellular and extracellular factors TGF-β, MYC, and IFN-γ may modulate the expression of PD-L1 downstream of YY1 control [13,85,133]. Several studies have shown that YY1 acts as a suppressor to IFN-γ through direct interaction of the IFN-γ promoter at several DNA binding sites [3,134,135]. YY1 represses the factor TGF-β through the inhibition of its immediate early genes, including plasminogen activator inhibitor 1 gene (PAI-1) and the inhibitor of differentiation (Id1) gene [63]. Recently, TGF-β has been a novel target for PD-L1 through its signaling in the TME and its implications with the PD-1/PD-L1 signaling pathway [136,137]. Additionally, downstream of TGF-β, PD-L1 expression is induced through signal transducer and activator of transcription 3 (STAT3) signaling [34,138]. Thus, the regulation of TGF-β by YY1 in certain contexts may act as another mechanism of YY1 transcriptional regulation of PD-L1. While further research is necessary to confirm the relationship between YY1, TGF-β, and PD-L1, there is promising evidence that this connection could influence cancer immunotherapy. YY1 has been found to be an active component in the *MYC* transcription network and is also associated with inhibition of MYC function [139,140]. *MYC*, as a regulator of PD-L1, was found to bind directly to the PD-L1 promoter regions, upregulating PD-L1 in tumor cells and leading to a dampened immune response [35,141]. 

YY1 overexpression has been determined to activate several signaling pathways as observed in several cancers [142,143,144]. Further, activation of these signaling pathways may indirectly regulate PD-L1 transcription as well. YY1 has been determined to activate the phosphatidylinositol 3-kinase/protein kinase B (PI3K/Akt) pathway through several mechanisms including phosphatase and tensin homolog (PTEN) inhibition [145]. Similar data showed that loss of PTEN was significantly associated with the resistance phenotype in tumor samples following treatment with CTL4-A blockade [146]. Downstream of the PI3K/Akt, the mechanistic Target of Rapamycin (mTOR) is highly associated with PD-L1 expression in non-small-cell lung cancer models [147]. Therefore, there is a likely connection between YY1 and increased PD-L1 expression via stimulation of PTEN/PI3K/Akt/mTOR signaling as proposed by us previously [138,147,148]. Moreover, PI3K inhibitors have been shown to act synergistically with checkpoint inhibitor therapies [149]. In addition to PI3K/Akt signaling, YY1 may also indirectly regulate PD-L1 transcription via the regulation of interleukin 6 (IL-6) and STAT3. YY1 has been determined to positively regulate IL-6 through direct binding to its promoter region and activation of transcription [34,37]. Further, IL-6 positively regulates STAT3, and STAT3 activates PD-L1 transcription via direct binding [34,150]. Thus, IL-6 may be an important factor in the regulation of PD-L1 by YY1. Furthermore, YY1 plays a dual role in regulating IFN-γ both positively and negatively in different cancers [34,151,152]. Hays and Bonavida [34] and Bellucci et al. [153] previously proposed that YY1 indirectly modulates PD-L1 expression through IFN-γ-induced activation of janus kinase-1 (JAK1), JAK2, and STAT1. In a recent study on ovarian cancer, it was similarly shown that IFN-γ may induce PD-L1 expression through JAK1, interferon regulatory factor 1, and STAT1-dependent signaling [154]. However, further experiments directly connecting YY1 to the IFN-γ/JAK1/STAT1-mediated regulation of PD-L1 are required to truly illustrate this proposed relationship. 

**Figure 3:**
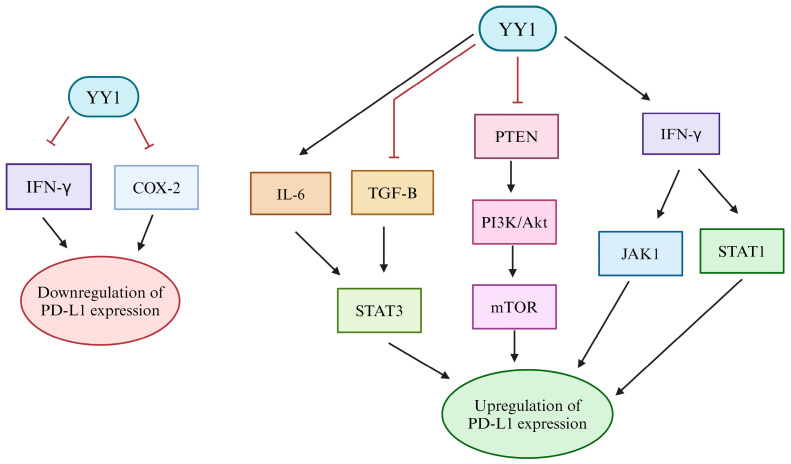
Upregulation and downregulation of PD-L1 by YY1 through various factors. YY1 indirectly regulates the expression of PD-L1 through several factors. YY1 plays a dual role in positively or negatively regulating IFN-γ in different cancers. The downregulation of PD-L1 is achieved when YY1 inhibits IFN-γ or COX-2. YY1 activation of IFN-γ leads to the activation of the JAK1 and STAT1 pathways, contributing to the upregulation of PD-L1 expression. Moreover, YY1 inhibition of PTEN activates the PI3K/Akt pathway, and ultimately, mTOR upregulation increases the expression of PD-L1. IL-6 and TGF-β are activated and inhibited, respectively, by YY1, leading to STAT3 upregulation and increased PD-L1 expression.

Cyclooxygenase 2 (COX-2), an enzyme associated with inflammatory responses, is another mediator in the regulation of PD-L1 by YY1. YY1 binds directly to the COX-2 promoter, positively regulating its expression in inflammatory contexts [155,156]. In vitro, inhibition of COX-2 was shown to downregulate PD-L1 expression in melanoma cells [157]. More recent evidence shows that COX-2 inhibitors can decrease the expression of PD-L1 in colon tumor cells [158]. COX-2 expression is also correlated with PD-L1 expression in breast cancer [159] and TAMs [160]. Its effect on PD-L1 may be traced to its known ability to activate STAT3 and NF-κB pathways, which are known regulators of PD-L1 [34,161,162,163]. STAT3 is a transcription factor that mediates cellular responses to various growth factors and cytokines [164,165,166,167]. In the context of cancer, its dysregulation can contribute to tumor progression due to its regulatory role in gene expression of cell differentiation, growth, and inflammation [168,169,170] (Figure 3).

### 4.2. Epigenetics

The most common epigenetic regulations of PD-L1 are DNA methylation and histone acetylation and methylation [171,172,173]. The enhancer of zeste homolog (EZH2) has been reported to be involved in the chromatin remodeling of PD-L1 via histone methylation [171,174,175]. EZH2 has also been shown to interact with YY1 in various pathways, including silencing tumor-suppressive miRNAs and other pro-tumorigenic properties [56,65]. Increasing immunotherapy effects, the STAT family targets PD-L1 through epigenetic regulation. Particularly, the small molecule Stri-201 inhibits PD-L1 expression [176,177]. Further mediations of STAT3 activity led to a reduction in PD-L1 such as eukaryotic initiation factor 4F (EIF4F) and HDAC6 [178,179]. Verheul et al. revealed that YY1 represses factors within the STAT family, particularly mammary gland factor (MGF) and STAT5A in mammary epithelial cells [41,81]. 

**Figure 4:**
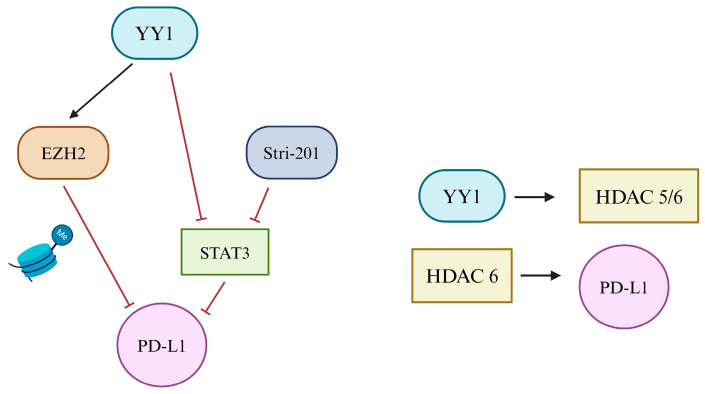
YY1 regulation of PD-L1: Epigenetic. Epigenetic regulation of PD-L1 through YY1 is achieved through various proposed mechanisms and relationships. YY1 directly activates EZH2 and, through histone methylation, inhibits PD-L1. Furthermore, YY1 and Stri-201, known inhibitors of STAT3, may indirectly downregulate PD-L1 expression. Next, YY1 has been found to interact with both HDAC5 and HDAC6 in many cancer cells. HDAC6 has a positive relationship with PD-L1, leading to the proposed mechanism between these factors.

Interestingly, several HDACs are implicated in PD-L1 regulation, and several HDAC inhibitor therapies have been shown to promote an anti-cancer effect, in part, due to modification of PD-L1 expression [180,181,182]. In B-cell lymphoma, inhibition of HDAC3 was shown to upregulate PD-L1 transcription [183], and in another study on lung cancer in mice, inhibition of HDAC6 was shown to decrease PD-L1 mRNA and protein levels [184]. Furthermore, YY1 has been confirmed to regulate and interact with several HDACs [72,185]. In acute myeloid leukemia (AML) cells, YY1 was revealed to bind and interact with HDAC1/3 [185]. In ovarian cancer, HDAC1, HDAC5, and HDAC6 were observed to be increased in cells overexpressing YY1. Additionally, YY1 was found to interact with both HDAC5 and HDAC6, though the interaction between YY1 and HDAC5 was found to be more robust [186]. While the ability of YY1 to epigenetically regulate PD-L1 warrants further investigation, YY1 may likely regulate PD-L1, in part, through acetylation via the regulation of HDACs (Figure 4).

### 4.3. Post-Transcriptional

YY1 is known to regulate the activity of several miRNAs upstream of PD-L1. YY1 upregulates PD-L1 expression through the inhibition of p53 [138,187]. Specifically, its transcriptional activity, acetylation, ubiquitination, and stability are regulated by YY1 [138,188,189]. In addition, p53 negatively regulates PD-L1 expression via interaction with miR-34a, and in non-small-cell lung cancer (NSCLC) cell models, miR-34a directly binds and downregulates PD-L1 [187]. The p53/miR-34/PD-L1 pathway is associated with tumor immune evasion, and YY1 regulation upstream of this pathway is likely a primary driver of this effect.

**Figure 5:**
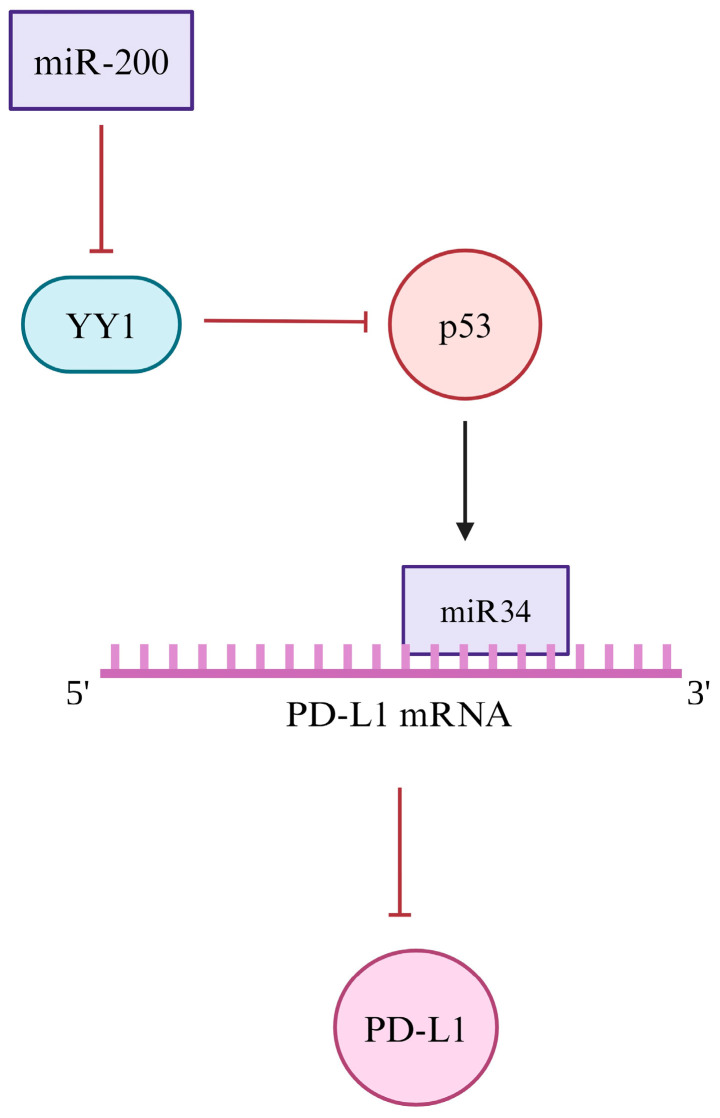
Post-translational regulation of PD-L1 expression. YY1 regulates PD-L1 via a post-translational mechanism. Firstly, miR-200 is negatively correlated with YY1, suggesting that miR-200 may decrease YY1 expression. YY1 is a known factor that inhibits p53, and p53 upregulates miR-34a. Ultimately this relationship leads to PD-L1 inhibition, and it is important to further identify ways to target PD-L1.

In addition, miR-200 contains a promoter sequence and binding site for YY1. YY1 expression is negatively correlated with miR-200, indicating downregulation upon binding [190,191]. A recent study by Zhang et al. [192] highlighted the use of miR-200 as an upstream target for PD-L1 in lung cancer prevention. This is due to the anti-tumor immunity effects of miR-200 and the reduction in PD-L1 expression [192]. Therefore, there is a potential correlation between YY1’s negative relationship with miR-200 which would ultimately modulate PD-L1 expression (Figure 5).

### 4.4. Post-Translational

Glycosylation, ubiquitination, sumoylation, and acetylation are all important factors in the stability of the PD-L1 protein. Post-translational modifications (PTMs) of PD-L1 lead to immune resistance, which modulates immunosuppression in cancer cells [133,193,194]. Generally, phosphorylation of PD-L1 promotes its degradation via the proteasome [133,194]. YY1 may indirectly mediate PD-L1 degradation via post-translational modification by glycogen synthase kinase 3 beta (GSK3β), a molecule that has been observed to be downstream of YY1 [194,195]. Specifically, YY1 has been shown to indirectly regulate the phosphorylation of GSK3β via several pathways [196]. In colorectal cancer, YY1 was shown to transcriptionally regulate transmembrane protein 97 (TMEM97), leading to downstream modification of GSK3β via phosphorylation [196]. Additionally, YY1 has been demonstrated to negatively regulate poly (ADP-ribose) polymerase (PARP) [195]. Later studies revealed that treatment of tumors with PARP inhibitors upregulated PD-L1 through suppression of GSK3β [195]. GSK3β is able to phosphorylate PD-L1, leading to proteasomal degradation [195]. Further, glycosylation of PD-L1 inhibits GSK3β binding and phosphorylation-dependent degradation. Therefore, only unglycosylated PD-L1 may be degraded in the aforementioned manner. 

Two other relevant molecules may also be linked to PD-L1 post-translational regulation. IL-6 and downstream-stimulated JAK1 are molecules downstream of YY1 also found to be implicated in PD-L1 phosphorylation. Differently from GSK3β, IL-6 and JAK1 are able to promote phosphorylation of PD-L1 at Tyr112, leading to overall PD-L1 stabilization [197]. Chan and colleagues determined that phosphorylation at this site leads to the recruitment of an N-glycosyltransferase, promoting glycosylation of PD-L1 and its stabilization. Overall, the various proposed mechanisms outlined above by which PD-L1 is regulated are summarized in Table 1.

## 5. Implications of YY1-Mediated Regulation of PD-1/PD-L1 Axis in Immune Escape

Impeding immune recognition is a crucial factor in tumor proliferation and cancer cell survival [198,199]. The activation of PD-1 by PD-L1 plays an important role in immunosuppression [200,201]. The PD-1/PD-L1 axis plays a large role in establishing an immune-inhibitory TME, shielding tumor cells from immune cell-mediated cell death [200,202,203,204]. In several solid cancers, the expression of PD-L1 on tumor cells is associated with heightened tumor aggressiveness and poor prognosis [203,205]. Upon activation of the PD-1/PD-L1 signaling pathway, several immune T-cell functions are inhibited, including antigen-specific T-cell activation, T-cell proliferation, and overall effector function [206,207]. Similarly, the transcription factor YY1 has been extensively studied and has been linked to pro-cancer activity in several cases [55,175]. As previously discussed, there are several mechanisms of PD-L1 regulation, with YY1 of specific interest due to its extensive oncogenic properties. As discussed above, we have outlined several mechanisms of regulation of PD-L1 by YY1. Overall, the upregulation of PD-L1 expression via YY1 through transcriptional, post-transcriptional, epigenetic, and post-translational regulation likely enhances the immune evasion of cancer cells. 

**Figure 6:**
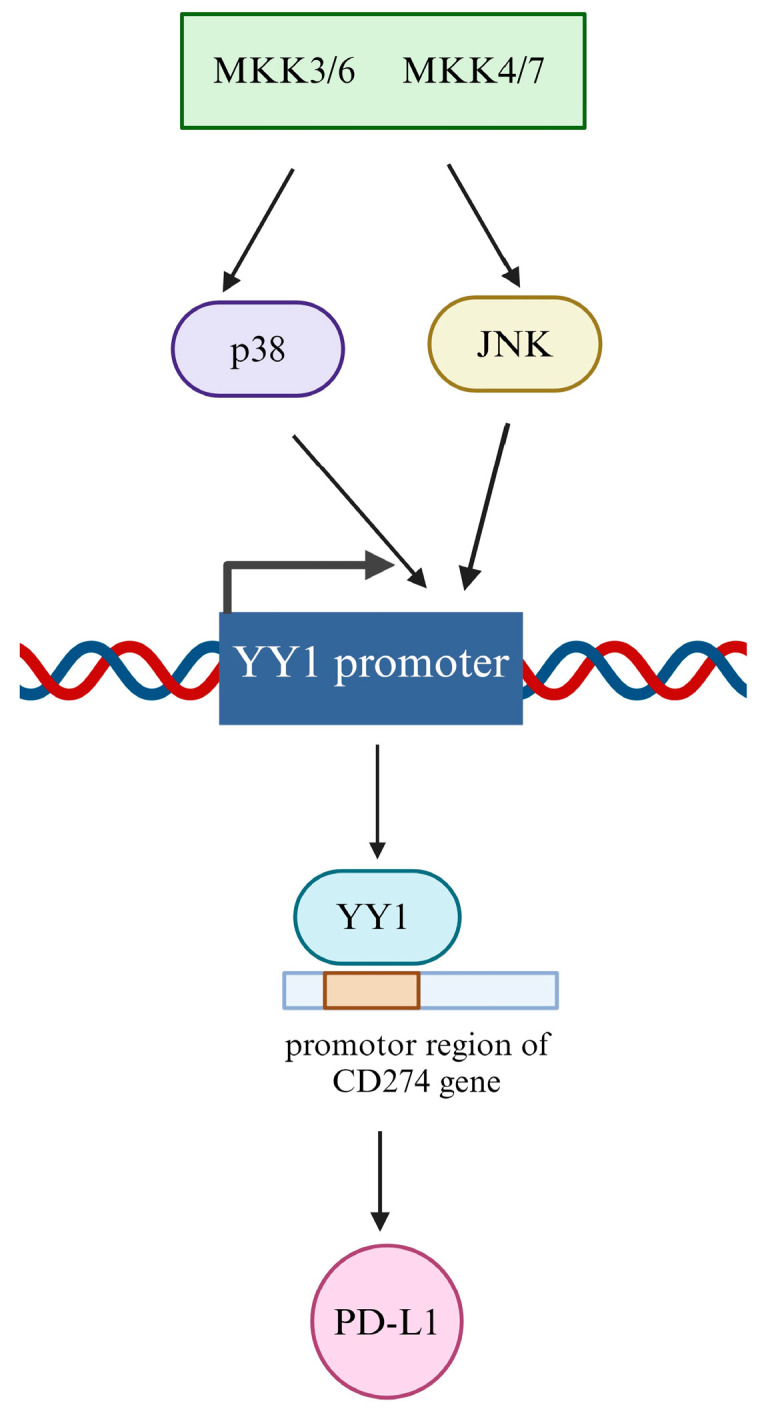
YY1 regulation of PD-L1: Transcriptional. YY1 regulates PD-L1 transcriptionally through binding to the promoter region found on the PD-L1 *CD274* gene. It is also regulated via the p38/MAPK/JNK pathway which upregulates YY1. This causes increased YY1 binding to the PD-L1 promoter and an increase in PD-L1 expression. This interaction leads to cancer progression and T-cell exhaustion of tumor-infiltrating lymphocytes. The red and blue lines represent the DNA helix.

In addition, YY1 is implicated with PD-1 upregulation in chronically stimulated T cells [196]. Balkhi and colleagues demonstrated that YY1-related signaling plays a significant role in upregulating checkpoint inhibitors such as PD-1, downregulating important cytokines, and contributing to T-cell exhaustion. Further, YY1 regulation of PD-1 is tied to tumor-infiltrating lymphocyte exhaustion [102,103]. T-cell exhaustion also plays a role in the progression of several cancer types and is a promising target for novel immunotherapies [101,201,208]. Furthermore, Balkhi et al. [102,107] found that in human melanoma cells, the activation of the p38/mitogen-activated protein kinase (MAPK)/jun N-terminal kinase (JNK) pathway leads to increased YY1 expression. YY1 then binds to the PD-1 promoter, ultimately leading to transcriptional upregulation. Thus, this pathway stimulates PD-1 upregulation via increased YY1 expression, leading to the exhaustion of tumor-infiltrating T lymphocytes and cancer progression (Figure 6).

**Figure 7:**
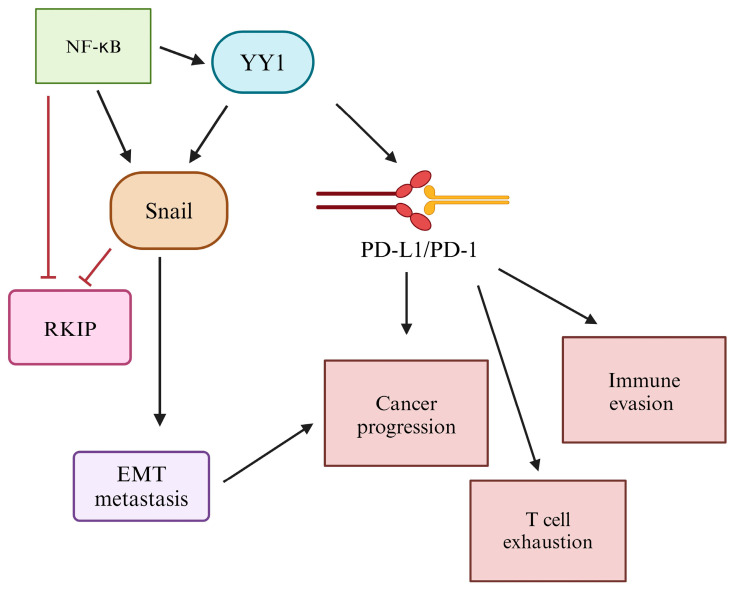
YY1-mediated regulation of PD-L1 and EMT. YY1 is an important therapeutic target due to its role in the regulation of the EMT and PD-L1. Through the NF-κB/Snail/YY1/RKIP loop, YY1 can be targeted to modulate the EMT, an important factor for immune escape in cancer cells. Additionally, YY1 upregulates PD-L1, leading to an increase in the PD-L1/PD-1 interaction. This leads to T-cell exhaustion, immune tolerance of tumor cells, and cancer progression.

Overexpression of YY1 is also correlated with immune resistance through the regulation of EMT. In the NF-κB/Snail/YY1/RKIP loop that regulates EMT, YY1 upregulates Snail, and in turn, Snail represses the immune surveillance gene product RKIP [206,207]. Further, the anti-apoptosis properties of YY1 via Fas, TRAIL, and DR5 regulation, as previously discussed, present further mechanisms of immune resistance as a result of YY1 overexpression. Previous studies have also suggested a high correlation between immune evasion due to EMT dysfunction and high PD-L1 expression in multiple cancers [209,210]. A study conducted by Kim et al. found that Snail expression was positively correlated with PD-L1 levels [211]. This relationship between EMT dysregulation and increased PD-L1 expression for immune evasion supports the importance of YY1 as a therapeutic target due to its critical role in the regulation of both EMT and PD-L1 (Figure 7).

The heterogeneous nuclear ribonucleoprotein L (HnRNP L), a protein involved in the regulation of mRNA splicing, is reported to be linked to cancer resistance and immune escape in prostate cancer. HnRNP L’s pro-cancer effects have been determined to be primarily caused by the stimulation of the YY1/PD-L1 axis [115]. It was shown that HnRNP L increases the expression of PD-L1 through the stabilization of YY1 mRNA via a direct binding mechanism. This stimulation of the YY1/PD-L1 axis inhibited Jurkat T-cell-mediated ferroptosis [212], promoting immune escape in prostate cancer cells. Overall, the inhibition of HnRNP L amplifies anti-tumor immune activity and is important in sensitizing cancer cells to anti-PD-1 therapy.

Tang et al. [213] outlined the role of the YY1–fibrinogen-like protein 1 (FGL1)–MYH9 axis in immune regulation. As a promising potential therapeutic target for lung cancer, researchers found that FGL1 expression was a promoter of the immune cytokine IL-2 secretion and T-cell apoptosis. They also discovered that FGL1 expression was positively associated with PD-L1 in lung adenocarcinoma (LUAD). A novel immunosuppressive agent to enhance the signal axis of FGL1/PD-L1 (FP) was developed by displaying FP on small extracellular vesicles (sEVs). This enhances axis expression and, in turn, increases immune inhibition [214]. Thus, it is proposed that PD-L1 may be coexpressed with FGL1 in tumor immunity as regulators [213]. 

In the context of NSCLC and LUAD, researchers Liu et al. [215] explored the resistance to the common treatment of epidermal growth factor receptor tyrosine kinase inhibitors (EGFR-TKIs) and their implications with PD-L1 and YY1. They found that NADPH oxidase 4 (NOX4) and YY1 expressions were positively linked to acquired resistance to EGFR-TKIs, while PD-L1, a downstream NOX4 target, ultimately contributed to immune escape in cancer cells. They further found that NOX4 induced YY1 expression, leading to YY1-mediated transcriptional activation of the factor interleukin 8 (IL-8). In vivo, NOX4, YY1, and IL-8 levels were shown to be heavily correlated with PD-L1 expression. It was determined that the NOX4/YY1/IL-8/PD-L1 signaling pathway is significantly implicated in NOX4-mediated therapy resistance and LUAD immune escape [215].

In summary, the various mechanisms by which PD-L1 is upregulated via YY1 contribute to heightened immune evasion. While PD-L1 is regulated by other transcription factors and mechanisms beyond YY1, the overexpression of YY1 does putatively lead to augmented immune evasion through multiple avenues, making it a promising therapeutic target via the several methods outlined in this section [211,212]. 

## 6. Bioinformatics Analysis of the Pathway Activity Scores (PASs) between High and Low YY1 and PD-L1-Expressing Tumors

**Figure 8:**
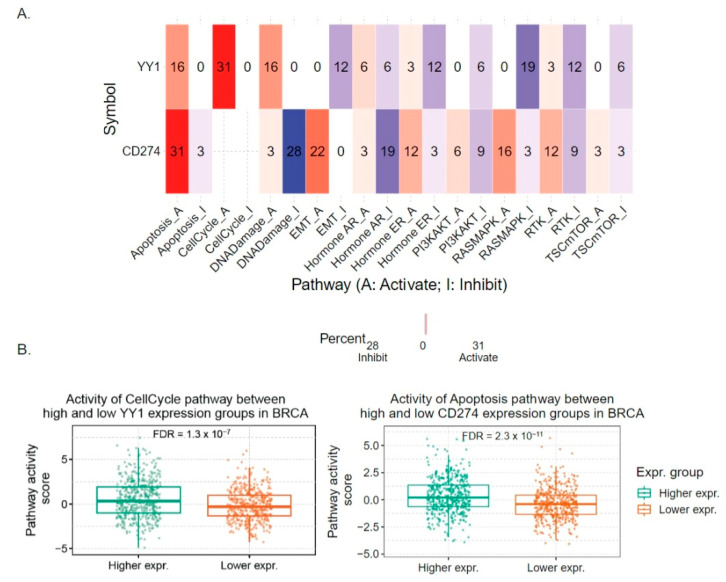
Bioinformatic analysis of PAS between high- and low-YY1 (or *CD274*)-expressing tumors (**A**) The heat map shows the percentage of cancers in which the YY1 and *CD274* (PD-L1) genes have an activating (red) or inhibitory (blue) effect (FDR ≤ 0.05) on 10 cancer-related pathways, across 32 TCGA cancer types. The number in each cell indicates the corresponding percentage. (**B**) Two examples of the activity of the Cell Cycle pathway between high- and low-YY1-expressing breast cancers, as well as of the activity of the Apoptosis pathway between high- and low-*CD274* (PD-L1)-expressing breast cancers (TCGA-BRCA).

To highlight the critical importance of targeting both YY1 and the PD-L1/PD-1 axis in several human cancers, we explored the differences in YY1 and *CD274* gene expressions between pathway activity groups (activation and inhibition), as defined by their median pathway scores (Supplementary Table S1 and Figure 8). 

To this end, we used reverse-phase protein array (RPPA) data from the cancer proteome atlas (TCPA, https://tcpaportal.org/tcpa/, accessed on 10 October 2023) to calculate the pathway activity scores (PASs) of 10 cancer-related pathways from 32 cancer types found in the cancer genome atlas (TCGA) project. RPPA is a high-throughput antibody-based technique during which proteins are extracted from tumor tissue or cultured cells, denatured by sodium dodecyl-sulfate (SDS), and printed on nitrocellulose-coated slides followed by an antibody probe. 

We paid attention to the following 10 cancer-related pathways: TSC/mTOR, receptor tyrosine kinase (RTK), RAS/MAPK, PI3K/AKT, Hormone ER, Hormone androgen receptor (AR), EMT, DNA Damage Response, Cell Cycle, and Apoptosis. 

RPPA data were median-centered and normalized by the standard deviation across all samples for each component to obtain the relative protein level. The pathway score was calculated as the sum of the relative protein level of all positive regulatory components minus that of negative regulatory components in a particular pathway, as previously described. 

Samples were then divided into two groups (high- and low-expressing tumors) by the median expression of each gene (*CD274* or *YY1*), and the difference in pathway activity score between the two groups was defined by the Student *t* test. *p* values were adjusted by false discovery rate (FDR), and FDR ≤ 0.05 was considered significant. The activating or inhibitory effect of YY1 or *CD274* on each pathway was applied as described in Ye et al. [63].

Our analysis showed that both genes are involved in the activation of apoptosis in pan-cancer, *CD274* at a much higher rate (16% of tumors for YY1 and 31% for *CD274*, respectively). YY1 was also shown to potentially activate the Cell Cycle pathway in 31% of all tumors and to inhibit the EMT, RTK, and RASMAPK pathways (in 12%, 12%, and 19% of all tumors, respectively). On the other hand, *CD274* mRNA expression was shown to have an inhibitory effect on the DNA Damage Response and Hormone AR pathways (28% and 19% of all tumors, respectively) and to activate the EMT, Hormone ER, RASMAPK, and RTK pathways (in 22%, 12%, 16% and 12% of all cancer types, respectively). 

We also examined the potential effect of each gene in specific tumor types. For example, in breast cancer, our analysis showed that tumors expressing high levels of YY1 had higher Cell Cycle PASs compared to those expressing low YY1 mRNA levels. Similarly, breast tumors expressing high levels of *CD274* had higher Apoptosis PASs compared to those expressing low *CD274* levels (Figure 8).

## 7. Therapeutic Targeting of YY1 for Immune Checkpoint Blockade

Due to YY1’s role in tumor progression and development, its inhibition may promote apoptosis and chemo-response, enhance immune CD8^+^ T-cell activity, and block tumor growth. Specific YY1-targeted therapies include the use of RNA interference, gene editing, and small-molecule inhibitors [141]. As outlined previously, inhibition of YY1 can indirectly downregulate PD-L1 through several mechanisms. The dual inhibition of YY1 and downstream PD-L1 may be an effective means of therapeutic targeting. It has been found that in melanoma patients, inhibiting YY1 restores downstream Fas- and TRAIL-mediated apoptosis [60,67,107]. Moreover, utilizing miRNAs to downregulate YY1 expression led to beneficial outcomes in melanoma patients due to the inhibition of the EMT [107,216]. More recently, YY1 regulation has been linked to the overexpression of several anti-apoptotic factors including Bcl-2, Bcl-xL, Mcl-1, and survivin [59,217,218,219,220]. Within the NF-κB/Snail/YY1/RKIP loop regulating the EMT, an inverse relationship between YY1 and RKIP exists, suggesting an additional potential source for indirect YY1 inhibition [74,221]. 

Nitric oxide (NO) has also been identified as an inhibitor of YY1, leading to suppressed EMT. This inhibition also sensitizes tumor cells to immunotherapies, including Fas-, TRAIL-, and anti-PD-L1-dependent treatments [67,107,222,223]. YY1-binding activity is inhibited via redox regulation by NO and occurs through thiol modification and disruption of YY1 activity [83]. Thus, targeting NO and blocking YY1 would allow for the downregulation of PD-L1 and the reversal of immune resistance. For example, in malignant melanoma, it was found that YY1 plays a critical role in apoptosis resistance, metastasis promotion, and the regulation of immune response [107,224,225,226]. This is achieved through its regulation of PD-1 and lymphocyte-activation gene 3. Inhibition of YY1 through NO allows melanoma cells to be more responsive to immunotherapies including anti-PD-L1-dependent treatment, further allowing anti-PD-L1 therapy to attack the cancer. NO is also known to play a role in the NF-κB/SNAIL/YY1/RKIP/PTEN loop in cancer cells through the repression of SNAIL. NO indirectly blocks YY1, which leads to chemotherapy sensitization. Additionally, in a study by Wang et al. [227] on skeletal myogenesis, the researchers discovered that YY1 serves as a transcriptional target of NF-κB, with NF-κB found to exert positive regulation of YY1 [227]. This is achieved through transcriptional regulation with NF-κB directly binding to the YY1 promoter, and it is not specific to cell types. Therefore, this suggests that inhibiting NF-κB could serve as another potential target by inhibiting YY1 expression on a transcriptional level.

The current development of small-molecule inhibitors of YY1 presents another promising method of YY1 inhibition [142,228,229]. Small-molecule inhibitors are chemical compounds that target specific proteins such as YY1 and modify their function [145]. The FDA has approved 89 small-molecule-targeted drugs for anti-tumor therapies and many more are currently undergoing clinical trials [75,142]. Examples include NO donors, small interfering RNA (siRNA) YY1, proteasome inhibitors, and inhibitors of activated survival pathways [75,81]. By working to directly or indirectly inhibit YY1 activity, small-molecule inhibitors are promising in achieving PD-1 inhibition [141,230]. 

A recent study by Gu et al. outlined a new potential immunotherapy via the blockage of PD-L1 [231]. The small-molecule antagonist to PD-L1, pentamidine, may be involved in inhibiting PD-1-to-PD-L1 interaction. Mechanistically, this is achieved through direct binding to the PD-L1 protein, promoting T-cell activity. The mediated cytotoxicity is permeated through the promotion of the factors IFN-γ and TNF-α [231]. YY1 has known implications with these factors as it is responsible for the inhibition of IFN-γ [134,135]. Additionally, the axis of TNF-α/NF-κB/YY1 highlights the relationship of TNF-α as an enhancer of YY1 expression [232,233,234,235]. Therefore, further research on the synergistic effects of YY1 blockers along with the PD-L1 blocker pentamidine could shed light on a more robust immune attack against cancer cells. The specific implications between YY1 and pentamidine would need to further be evaluated. 

The development of a small synthetic small-molecule-specific YY1 inhibitor (Inh-YY1) can inhibit the YY1-DNA binding activity due to repressor activity on the *Fas* gene activation of the *MDR1* gene [236]. The findings with drug-resistant pediatric lymphoblastoid cell lines demonstrated that treatment with Inh-YY1 reverted resistance when used in combination with chemotherapeutic drugs. However, further research is needed to develop tumor-cell-specific Inh-YY1, possibly through the use of Inh-YY1–antibody conjugates and nanotechnologies, for clinical applications [236].

Additionally, the compound isorhamnetin (ISO) was found to target YY1 in hepatocellular carcinoma (HCC) in a study conducted by Liu et al. [237]. In slowing the progression of HCC, anti-PD-L1 antibodies are normally used as immunotherapy; however, the EMT of these tumor cells may create an immunosuppressive environment and promote tumor migration. YY1 is an important regulator of the EMT and migration of HCC cells, and, thus, can be targeted to inhibit HCC tumor growth through ISO. The development of a combined nanoparticle-based therapy (HMSN-ISO@ProA-PD-L1 Ab) has revealed promising results, by both releasing ISO in a controlled manner and reducing the immunosuppressive properties of PD-L1 in the TME [237]. 

Antisense oligonucleotides (ASOs) are chemically synthesized oligonucleotides that bind to a target RNA strand and modulate its function through various mechanisms [238,239]. There has been increasing interest in the therapeutic applications of ASOs with the approval of several ASO drugs in late-stage clinical studies [238,240]. ASOs are generally 12–30 nucleotides long and promote RNA cleavage and degradation or block protein translation [238,239]. Occupancy-only mechanisms involve the ASOs binding to RNA to block translation and replication. The more common type of ASOs involves RNA degradation mechanisms, where ASOs are designed to promote RNA cleavage via RNase H1 or argonaute 2 [238,240]. The limitations of the therapeutic use of ASOs are tied to the rapid degradation of unmodified RNA or DNA oligonucleotides in biological matrices. Therefore, chemical modifications are necessary to ensure their role as a viable therapeutic agent [238,241,242,243]. Overall, the ability to target specific RNA sequences suggests a potential method of inhibiting YY1 expression at the RNA level but requires further research and development. 

Similar to the specificity achieved through the use of ASOs, another possible mechanism of YY1 inhibition is via CRISPR/Cas9 technologies [60,244]. With the use of guide RNAs (gRNAs), it is possible to directly target the YY1 gene for CRISPR/Cas9-mediated knockout. Once YY1 is targeted, Cas9 can cleave the DNA sequence and disrupt gene function. This inhibition of YY1 expression would inhibit the downstream effects of immune evasion by PD-L1 expression and other oncogenic properties associated with the overexpression of YY1 [60,102].

Epigenetic modifications have been known to play a role in the development of cancer; however, the development of novel drugs aims to reverse these modulations [245]. Thus, YY1 inhibition via targeting epigenetic changes is an attractive approach to consider. An approach aimed specifically at modifying epigenetic marks, called epidrugs, may be an effective novel therapeutic strategy. The use of epidrugs to target, reverse, or inhibit DNA methylation, acetylation, HDAC activation, transcription, and post-transcriptional activities can reduce chemoresistance in certain cancers [245]. MiRNAs are one form of epigenetic modulation, consisting of short strands of non-coding RNA that target and modulate mRNA expression post-transcriptionally. Several miRNAs have been found to have an association with YY1 expression in cancer [75,246]. In CRC, the overexpression of miR-7 is correlated with the inhibition of proliferation, induction of apoptosis, and induced cell arrest in G1 [75,247]. MiR-7 directly binds to YY1 and downregulates YY1 expression in CRC, ultimately inducing apoptosis [59,75,198]. Furthermore, studies focused on cervical cancer cases found that miR-181 negatively regulates YY1 expression and additionally inhibits tumor cell proliferation [248]. In esophageal squamous cell carcinoma, an association with miR-34A was found, and in cases of pancreatic cancer, miR-489 associations were noted [249,250]. Additionally, Yang et al. [246] reported that miRNAs can modulate YY1 gene expression through the association between miRNA-193a-5P and YY1. Thus, miRNAs offer another promising solution for the inhibition of YY1 and other tumorigenic effects.

In a study by Liu and colleagues [251] on glioblastoma patients, siRNA was tested as a possible approach for therapeutic interventions. A T7 peptide-decorated exosome (T7-exo) was used to efficiently target and deliver the siRNA to tumor cells both in vitro and in vivo [251]. The in vitro experiments found that the T7-siYY1-exo complex was able to enhance chemoradiotherapy sensitivity and reverse therapeutic resistance while also significantly improving the survival time of the mice with glioblastoma [251]. YY1-siRNA in combination with a tumor-specific delivery system such as a peptide-decorated exosome is a possible mechanism of YY1 inhibition and requires further exploration. 

**Figure 9:**
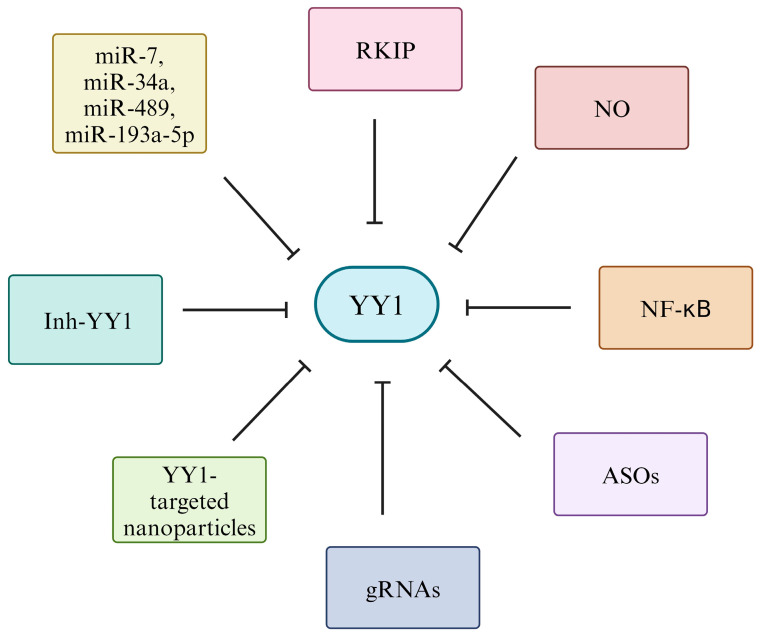
Various targets that lead to the inhibition of YY1. Through the inhibition of YY1 and PD-L1 downregulation, oncogenic effects may decrease. Several targets are identified to achieve negative regulation of YY1. RKIP is a target that has a known inverse relationship with YY1 through the NF-κB/Snail/YY1/RKIP loop. NO is also implicated with the inhibition of YY1 through the repression of SNAIL, while NF-κB is also a promising factor to consider in the context of the NF-κB/SNAIL/YY1/RKIP/PTEN loop. ASOs also provide specificity in inhibiting YY1 through their ability to bind to specific RNA target sequences. Next, the use of miR-7, miR-34a, miR-489, or miR-193a-5p may have the ability to regulate YY1 gene expression negatively. The use of gRNAs through CRISPR/Cas9 may block YY1 gene function through disruption of the DNA sequence. The use of a novel synthetic small-molecule inhibitor, Inh-YY1, may directly inhibit the DNA-binding activity of YY1. Finally, YY1-targeted nanoparticle delivery is a promising selective method of YY1 inhibition. It is proposed to be used in combination with other methods of YY1 inhibition as previously mentioned.

The development of YY1-targeted nanoparticles poses another potential mechanism for delivering targeted treatment. The clinical uses of targeted nanoparticles are still being investigated, but many nanoparticles are designed to deliver immunotherapeutic agents directly to T cells [252,253]. A study by Riley et al. [252,253] targeted TGF-βR1 inhibitors to T cells via nanoparticles and extended the survival in a mouse model of CRC when compared to the use of free drugs. The use of a nanoparticle drug delivery system increased the proportion of tumor-infiltrating CD8^+^ T cells and sensitized tumors to anti-PD-1 therapy [252,253]. In combination with the previously discussed methods of YY1 inhibition, YY1-targeted nanoparticle delivery is a potential method of selective inhibition of YY1 to reverse immune evasion and resistance (Figure 9).

The above-discussed methods and potential routes for YY1 inhibition are promising therapeutic targets for tumor cells. However, blocking PD-L1 via YY1 inhibition poses the need for a therapy that is specific and selective to tumor cells to ensure no adverse effects on healthy cells. Achieving this will require a combination of strategies, technologies, methods, and further investigations. The increased immune evasion associated with the overexpression of YY1 and its regulation of PD-L1 among other cellular pathways strongly supports targeting YY1 for inhibition. Doing so may reverse the oncogenic phenotype of tumor cells. Each of these proposed methods of selectively targeting YY1 for inhibition requires further research and exploration for efficacy.

## 8. Overall Perspectives and Future Directions

The findings as described above have illustrated several likely mechanisms for the upregulation of PD-L1 in cancer by YY1. We have also specifically highlighted several putative and previously observed means of transcriptional, post-transcriptional, epigenetic, and post-translational regulations of PD-L1 by YY1. YY1 has been shown to regulate PD-L1 transcription both directly and indirectly through stimulation of several factors including MYC, TGF-β, IFN-γ, and IL-6 [34,35,36,37,38,39]. Furthermore, YY1 regulates PD-L1 through activation of the transcriptional pathways STAT, JAK, PI3K/Akt, and NF-κB. Another inflammatory enzyme, COX-2, has also been identified as a potential downstream target of YY1 and a positive regulator of PD-L1 [155,157,158,222]. Additionally, YY1 may indirectly control PD-L1 epigenetically through the regulation of HDACs and STAT downstream small molecules [176,180,184]. Some miRNAs have also been found to be involved in YY1 regulation of PD-L1, including miR-34 and miR-200. Finally, YY1 is implicated in PD-L1 degradation or stabilization through post-translational modification. YY1 regulates PD-L1 through indirect control of both GSK3β and JAK1, leading to its phosphorylation and either stabilization or degradation [194,195,196,197].

While immune checkpoint inhibitors that target PD-L1 have been promising in treating cancer, many patients are not responsive to these therapies. Thus, it is important to further understand its implications and associated pathways that play a role in therapy resistance [111]. There is significant current evidence regarding the impact of YY1 regulation of PD-L1 in several cancers. Multiple studies have implicated the YY1/PD-L1 axis in immune escape in melanoma, NSCLC, liver cancer, and lymphoma [47,156]. It has been determined that YY1 is an important regulator of PD-L1 during T-cell exhaustion and promotes immune escape in prostate cancer [115,208,212]. YY1 is also implicated in the resistance of immune checkpoint therapies, and it can disrupt immunotherapy treatments through disruption of the PD-1/PD-L1 axis [156,213]. Additionally, YY1 is linked to tumor immune evasion through the upregulation of PD-L1 through regulation of the p53/miR-34/PD-L1 pathway [187]. Therefore, we have proposed that YY1 targeting is a beneficial therapeutic strategy for circumventing resistance to conventional immunotherapies. Further, we have illustrated several novel ways of targeting both YY1 and PD-L1 in hopes of reversing resistance and sensitizing tumor cells to current treatments [59,142,231,237]. Though findings indicate a strong connection between YY1 and PD-L1, the complete landscape of YY1 regulation of PD-L1 requires further investigation. Future studies on other shared pathways are needed to strengthen our understanding of the YY1/PD-L1 axis as well as the various proposed mechanisms of YY1 inhibition.

Despite the various potential methods of YY1 inhibition, there remain challenges in the development of a viable therapeutic agent. The most significant being the necessity of selectivity and specificity in inhibition. In order to reduce the negative consequences of a potential therapeutic, a YY1 inhibitor must selectively target YY1 expression in tumor cells, leaving healthy cells untouched. To combat this, it is likely that a selective nanoparticle, antibody, or other technology will be needed in combination with the YY1 inhibitor to target YY1 expression solely in tumor cells. This specificity remains a crucial factor for the various methods of cancer treatment and will require further investigation of its possible avenues.

This review highlights the regulation of PD-L1 expression via YY1 or other crosstalk pathways in cancer. Targeting YY1 will not only be an effective strategy for reducing PD-L1 expression in certain cancers but also for promoting various positive effects through interactions with other crosstalk pathways and downstream inhibition of several other known cancer-promoting gene products. Further research focused on identifying the connections between these crosstalk pathways, YY1, and PD-L1 is likely to yield promising results for the development of future therapies.

In conclusion, our study underscores the significance of YY1 as a master regulator in cancer immune evasion, intricately controlling PD-L1 expression through various transcriptional, post-transcriptional, epigenetic, and post-translational mechanisms. Targeting YY1 offers a multifaceted approach to not only disrupt the PD-1/PD-L1 axis but also attenuate other pathways contributing to tumor aggressiveness and therapy resistance. However, the development of specific YY1 inhibitors that selectively target tumor cells while sparing healthy tissues poses a substantial challenge and necessitates further research. This strategy, integrating selective YY1 inhibition with current immunotherapies, could potentially revolutionize the treatment paradigm for resistant cancers, ultimately improving patient outcomes.

## Figures and Tables

**Table 1:** Summarized molecular regulation of PD-L1 expression by YY1.

Type of Regulation	Modifier or Pathway Downstream of YY1	Effect on PD-L1	Reference
Transcriptional	YY1 direct binding to the PD-L1 promoter	Promotes Transcription	[132]
	Repression of TGF-β	Decreased expression	[63,136]
	Extension of *MYC*-related transcription factor networks	Increased expression	[80,81,82]
	Inhibition of MYC function	Decreased expression	[35,140,141]
	PI3K/Akt/mTOR activation via PTEN inhibition	Increased expression	[34,145,146,147]
	Positive regulation of IL-6/STAT3	Increased expression	[37,150,161,162,163]
	IFN-γ-induced activation of JAK1 and STAT1	Increased expression	[34,153,154]
	Increased COX-2 expression	Increased expression	[155,156,157,158,159,160]
Epigenetic	Increased HDAC6	Upregulation	[184,186]
	Interaction with EZH2	Upregulation	[56,65,171]
	Repression of STAT family proteins	Upregulation	[141,142]
Post-transcriptional	Inhibition of p53 and miR-34	Upregulation	[187,188,189]
	Downregulation of miR-200	Downregulation	[190,192]
Post-translational	Suppression of PARP/GSK3β	Degradation	[194,195,196]
	Stimulation of IL-6/JAK1	Stabilization	[37,197]

COX-2: cyclooxygenase 2; EZH2: enhancer of zeste homolog; GSK3β: glycogen synthase kinase 3 beta; HDAC: histone deacetylase; IFN-γ: interferon gamma; IL-6: interleukin 6; JAK: janus kinase; mTOR: mechanistic Target of Rapamycin; miR-200: microRNA-200; miR-34: microRNA-34; *MYC*: myelocytomatosis oncogene; p53: tumor protein p53; PARP: poly (ADP-ribose) polymerase; PD-L1: programmed death ligand-1; PI3k/AKT: phosphatidylinositol 3-kinase/protein kinase B; PTEN: phosphatase and tensin homolog; STAT: signal transducer and activator of transcription; STAT1: signal transducer and activator of transcription 1; STAT3: signal transducer and activator of transcription 3; TGF-β: transforming growth factor-β; YY1: Yin Yang 1.

## Supplementary Materials

The following supporting information can be downloaded at https://www.mdpi.com/article/10.3390/cancers16061237/s1, Supplementary Table S1. Reverse phase protein array data as shown in Figure 8. We investigated variations in the expression of the YY1 and *CD274* (PD-L1) genes across pathway activity groups, as defined by their median pathway scores. The bioinformatics analysis spanned 32 diverse cancer types, with a particular focus on the following 10 cancer-related pathways: TSC/mTOR, receptor tyrosine kinase (RTK), RAS/MAPK, PI3K/AKT, Hormone ER, Hormone androgen receptor (AR), EMT, DNA Damage Response, Cell Cycle, and Apoptosis.

## Abbreviations

AML	acute myeloid leukemia
AR	androgen receptor
ASO	antisense oligonucleotide
CAFS	cancer-associated fibroblasts
CD4^+^/CD8^+^ TILs	tumor-infiltrating CD4^+^/CD8^+^ T cells
COX-2	cyclooxygenase 2
CRC	colorectal cancer
CRHR2	corticotropin-releasing hormone receptor 2
CRPC	castration-resistant prostate cancer
CTLA-4	cytotoxic T-lymphocyte-associated protein-4
DC	dendritic cell
DR5	death receptor 5
EGFR-TKI	epidermal growth factor receptor tyrosine kinase inhibitor
EIF4F	eukaryotic initiation factor 4F
EMT	epithelial–mesenchymal transition
EZH2	enhancer of zeste homolog
FDA	Food and Drug Administration
FDR	false discovery rate
FGL1	fibrinogen-like protein 1
FP	FGL1/PD-L1
GSK3β	glycogen synthase kinase 3 beta
GRNA	guide RNA
HCC	hepatocellular carcinoma
HDAC	histone deacetylase
HnRNP L	heterogeneous nuclear ribonucleoprotein L
Id1	inhibitor of differentiation
IFN-γ	interferon gamma
IL-2	interleukin 2
IL-6	interleukin 6
IL-8	interleukin 8
Inh-YY1	YY1 inhibitor
ISO	isorhamnetin
JAK	janus kinase
JNK	jun N-terminal
LUAD	MYH9 lung adenocarcinoma
mAbs	monoclonal antibodies
MDSC	myeloid-derived suppressor cell
MGF	mammary gland factor
miRNA	microRNA
MOR	mu opioid receptor
mTOR	mechanistic Target of Rapamycin
NF-κB	nuclear factor kappa B
NK	natural killer
NO	nitric oxide
NOX4	NADPH oxidase 4
NSCLC	non-small-cell lung cancer
PAI-1	plasminogen activator inhibitor 1 gene
PARP	poly (ADP-ribose) polymerase
PAS	pathway activity scores
PD-1	programmed death-1
PD-L1	programmed death ligand-1
PD-L2	programmed death ligand-2
PI3k/AKT	phosphatidylinositol 3-kinase/protein kinase B
PTEN	phosphatase and tensin homolog
PTM	post-translational modification
RAS/MAPK	Ras/mitogen-activated protein kinase
RKIP	Raf kinase inhibitory protein
RPPA	reverse-phase protein array
RTK	receptor tyrosine kinase
sEVs	small extracellular vesicles
SDS	sodium dodecyl-sulfate
siRNA	small interfering RNA
Sp1	specificity protein 1
STAT	signal transducer and activator of transcription
T7-exo	T7 peptide-decorated exosome
TAM	tumor-associated macrophage
TCGA	the cancer genome atlas
TCPA	the cancer proteome atlas
TF	transcription factor
TGF-β	transforming growth factor-β
TIL	tumor-infiltrating lymphocyte
TME	tumor microenvironment
TMEM97	transmembrane protein 97
TNF-α	tumor necrosis factor-alpha
TRAIL	tumor necrosis factor-related apoptosis-inducing ligand
Treg	regulatory T cell
Ucn2	Urocortin-2
YY1	Yin Yang 1
