# Supplementary material for: Regulation of PD-L1 Expression by YY1 in Cancer: Therapeutic Efficacy of Targeting YY1
Source: Cancers (Basel). 2024 Mar 21;16(6):1237. doi: 10.3390/cancers16061237 (PMC10968822; doi:10.3390/cancers16061237)
Supplement: Supplementary file 1 [file cancers-16-01237-s001.zip › cancers-2840405-supplementary.pdf]

cancers

MDPI

Supplementary Table S1. Reverse phase protein array data as shown in Figure 8. We investigated variations in the expression of the YY1 and CD274 (PD-L1) genes across pathway activity groups, as defined by their median pathway scores. The bioinformatics analysis spanned 32 diverse cancer types, with a particular focus on the following 10 cancer-related pathways: TSC/mTOR, receptor tyrosine kinase (RTK), RAS/MAPK, PI3K/AKT, Hormone ER, Hormone androgen receptor (AR), EMT, DNA Damage Response, Cell Cycle, and Apoptosis.

cancertype	symbol	pathway	fdr	class	diff	entrez
ACC	CD274	Apoptosis	0.029056501	Inhibition	-1.364076106	29126
ACC	CD274	CellCycle	0.196818963	None	-1.038203629	29126
ACC	CD274	DNADamage	0.463102181	None	-0.530278631	29126
ACC	CD274	EMT	0.432251833	None	-0.525309434	29126
ACC	CD274	Hormone AR	0.396637205	None	-0.56081063	29126
ACC	CD274	Hormone ER	0.201520642	None	0.488352853	29126
ACC	CD274	PI3KAKT	0.474624621	None	-0.611499997	29126
ACC	CD274	RASMAPK	0.197994082	None	0.670859728	29126
ACC	CD274	RTK	0.807214684	None	0.101631198	29126
ACC	CD274	TSCmTOR	0.796399724	None	-0.127390385	29126
ACC	YY1	Apoptosis	0.252569435	None	0.924890577	7528
ACC	YY1	CellCycle	0.852335582	None	0.128376333	7528
ACC	YY1	DNADamage	0.51556399	None	0.543415436	7528
ACC	YY1	EMT	0.372072734	None	0.710938668	7528
ACC	YY1	Hormone AR	0.62473271	None	-0.316619853	7528
ACC	YY1	Hormone ER	0.252569435	None	-0.553326903	7528
ACC	YY1	PI3KAKT	0.62473271	None	0.46369036	7528
ACC	YY1	RASMAPK	0.348615239	None	-0.604628954	7528
ACC	YY1	RTK	0.51556399	None	-0.437471723	7528
ACC	YY1	TSCmTOR	0.852335582	None	0.06505651	7528

BLCA	CD274	Apoptosis	3.91E-10	Activation	1.0748183	29126
BLCA	CD274	CellCycle	0.450161165	None	0.153326754	29126
BLCA	CD274	DNADamage	0.00158051	Inhibition	-0.504028177	29126
BLCA	CD274	EMT	2.18E-08	Activation	1.90003154	29126
BLCA	CD274	Hormone AR	1.94E-08	Inhibition	-1.056321989	29126
BLCA	CD274	Hormone ER	0.071566249	None	0.141996316	29126
BLCA	CD274	PI3KAKT	0.450161165	None	-0.238899301	29126
BLCA	CD274	RASMAPK	0.76869089	None	-0.056477586	29126
BLCA	CD274	RTK	0.001300732	Inhibition	-0.783551594	29126
BLCA	CD274	TSCmTOR	0.75437894	None	-0.065215337	29126
BLCA	YY1	Apoptosis	0.186733956	None	0.267364435	7528
BLCA	YY1	CellCycle	0.000712178	Activation	0.660546668	7528
BLCA	YY1	DNADamage	0.026859908	Activation	0.373796819	7528
BLCA	YY1	EMT	0.026673242	Inhibition	-0.85994649	7528
BLCA	YY1	Hormone AR	0.418883561	None	0.163491369	7528
BLCA	YY1	Hormone ER	0.026673242	Inhibition	-0.179724574	7528
BLCA	YY1	PI3KAKT	0.304909711	None	-0.303896336	7528
BLCA	YY1	RASMAPK	0.010121283	Inhibition	-0.591927489	7528
BLCA	YY1	RTK	0.80046643	None	-0.057602498	7528
BLCA	YY1	TSCmTOR	0.418883561	None	-0.1441585	7528
BRCA	CD274	Apoptosis	2.29E-11	Activation	0.733962059	29126
BRCA	CD274	CellCycle	0.19325081	None	0.206067342	29126
BRCA	CD274	DNADamage	0.846297002	None	-0.025545197	29126
BRCA	CD274	EMT	0.039183204	Activation	0.455516675	29126

BRCA	CD274	Hormone AR	0.000059619	Inhibition	-0.921209739	29126
BRCA	CD274	Hormone ER	0.000026136	Inhibition	-1.278971172	29126
BRCA	CD274	PI3KAKT	0.039183204	Activation	0.394663828	29126
BRCA	CD274	RASMAPK	0.441104986	None	0.118724961	29126
BRCA	CD274	RTK	0.336638014	None	0.134571359	29126
BRCA	CD274	TSCmTOR	0.325806109	None	0.136040245	29126
BRCA	YY1	Apoptosis	8.35E-06	Activation	0.505750378	7528
BRCA	YY1	CellCycle	1.32E-07	Activation	0.739176774	7528
BRCA	YY1	DNADamage	0.282944663	None	0.227123437	7528
BRCA	YY1	EMT	0.340565381	None	-0.248130011	7528
BRCA	YY1	Hormone AR	0.810138724	None	0.106704056	7528
BRCA	YY1	Hormone ER	0.298757993	None	-0.407185001	7528
BRCA	YY1	PI3KAKT	0.810138724	None	0.048650371	7528
BRCA	YY1	RASMAPK	0.810138724	None	-0.038818641	7528
BRCA	YY1	RTK	0.810138724	None	-0.029273854	7528
BRCA	YY1	TSCmTOR	0.298757993	None	-0.164090049	7528
CESC	CD274	Apoptosis	0.353222236	None	0.34808516	29126
CESC	CD274	CellCycle	0.540729387	None	-0.193448655	29126
CESC	CD274	DNADamage	0.30901132	None	-0.558472562	29126
CESC	CD274	EMT	0.540729387	None	0.446571099	29126
CESC	CD274	Hormone AR	0.353222236	None	-0.423341779	29126
CESC	CD274	Hormone ER	0.565058826	None	-0.18503937	29126
CESC	CD274	PI3KAKT	0.808486886	None	0.109845664	29126
CESC	CD274	RASMAPK	0.615496682	None	-0.224402648	29126

CESC	CD274	RTK	0.906678158	None	0.034704581	29126
CESC	CD274	TSCmTOR	0.755113284	None	-0.106083183	29126
CESC	YY1	Apoptosis	0.157642448	None	-0.367475399	7528
CESC	YY1	CellCycle	0.72570602	None	-0.06135411	7528
CESC	YY1	DNADamage	0.106168186	None	-0.612593878	7528
CESC	YY1	EMT	0.72570602	None	-0.141552739	7528
CESC	YY1	Hormone AR	0.026741063	Inhibition	-0.764327085	7528
CESC	YY1	Hormone ER	0.157642448	None	-0.33304655	7528
CESC	YY1	PI3KAKT	0.157642448	None	0.610889666	7528
CESC	YY1	RASMAPK	0.42943173	None	0.269511435	7528
CESC	YY1	RTK	0.157642448	None	0.484376732	7528
CESC	YY1	TSCmTOR	0.20850631	None	0.306873715	7528
CHOL	CD274	Apoptosis	0.840751867	None	-0.086677098	29126
CHOL	CD274	CellCycle	0.599460611	None	0.427709875	29126
CHOL	CD274	DNADamage	0.840751867	None	0.13714724	29126
CHOL	CD274	EMT	0.840751867	None	-0.152470719	29126
CHOL	CD274	Hormone AR	0.840751867	None	0.129760745	29126
CHOL	CD274	Hormone ER	0.695686633	None	0.328901894	29126
CHOL	CD274	PI3KAKT	0.840751867	None	0.291034641	29126
CHOL	CD274	RASMAPK	0.599460611	None	0.698160084	29126
CHOL	CD274	RTK	0.599460611	None	0.536489324	29126
CHOL	CD274	TSCmTOR	0.840751867	None	0.200210653	29126
CHOL	YY1	Apoptosis	0.953409023	None	0.099339831	7528
CHOL	YY1	CellCycle	0.953409023	None	-0.057778528	7528

CHOL	YY1	DNADamage	0.953409023	None	-0.535563869	7528
CHOL	YY1	EMT	0.953409023	None	-0.592169619	7528
CHOL	YY1	Hormone AR	0.988209077	None	-0.005983232	7528
CHOL	YY1	Hormone ER	0.953409023	None	-0.159119828	7528
CHOL	YY1	PI3KAKT	0.953409023	None	-0.121247598	7528
CHOL	YY1	RASMAPK	0.953409023	None	0.426729765	7528
CHOL	YY1	RTK	0.953409023	None	0.31321196	7528
CHOL	YY1	TSCmTOR	0.953409023	None	-0.261752523	7528
COAD	CD274	Apoptosis	0.00326232	Activation	0.826552307	29126
COAD	CD274	CellCycle	0.291583724	None	0.243701262	29126
COAD	CD274	DNADamage	0.001122232	Inhibition	-0.566233037	29126
COAD	CD274	EMT	0.000894906	Activation	1.430275703	29126
COAD	CD274	Hormone AR	0.771108597	None	-0.04073036	29126
COAD	CD274	Hormone ER	0.023448449	Activation	0.249260265	29126
COAD	CD274	PI3KAKT	0.073787371	None	0.587659183	29126
COAD	CD274	RASMAPK	0.023448449	Activation	0.791796402	29126
COAD	CD274	RTK	0.00074059	Activation	0.728155661	29126
COAD	CD274	TSCmTOR	0.103839395	None	0.333188586	29126
COAD	YY1	Apoptosis	0.603217654	None	0.236914442	7528
COAD	YY1	CellCycle	0.512343808	None	0.247987417	7528
COAD	YY1	DNADamage	0.723240457	None	0.056598611	7528
COAD	YY1	EMT	0.723240457	None	-0.171203999	7528
COAD	YY1	Hormone AR	0.621529316	None	0.108913917	7528
COAD	YY1	Hormone ER	0.723240457	None	-0.038412752	7528

COAD	YY1	PI3KAKT	0.172248464	None	-0.58434037	7528
COAD	YY1	RASMAPK	0.037843751	Inhibition	-0.856314416	7528
COAD	YY1	RTK	0.022696262	Inhibition	-0.56430554	7528
COAD	YY1	TSCmTOR	0.440211613	None	-0.259774589	7528
DLBC	CD274	Apoptosis	0.032985601	Activation	1.22865819	29126
DLBC	CD274	CellCycle	0.194741806	None	-0.719759886	29126
DLBC	CD274	DNADamage	0.032985601	Inhibition	-1.516037254	29126
DLBC	CD274	EMT	0.93250603	None	-0.171200295	29126
DLBC	CD274	Hormone AR	0.168374349	None	-0.804072261	29126
DLBC	CD274	Hormone ER	0.043314521	Activation	0.474245505	29126
DLBC	CD274	PI3KAKT	0.93250603	None	-0.24722599	29126
DLBC	CD274	RASMAPK	0.985961305	None	-0.046697134	29126
DLBC	CD274	RTK	0.985961305	None	0.009000893	29126
DLBC	CD274	TSCmTOR	0.360432095	None	0.502439573	29126
DLBC	YY1	Apoptosis	0.77110014	None	-0.137081924	7528
DLBC	YY1	CellCycle	0.77110014	None	0.408716876	7528
DLBC	YY1	DNADamage	0.77110014	None	0.353365823	7528
DLBC	YY1	EMT	0.630671038	None	-0.561243045	7528
DLBC	YY1	Hormone AR	0.630671038	None	0.594203882	7528
DLBC	YY1	Hormone ER	0.630671038	None	-0.332164896	7528
DLBC	YY1	PI3KAKT	0.77110014	None	0.534401564	7528
DLBC	YY1	RASMAPK	0.77110014	None	0.160722184	7528
DLBC	YY1	RTK	0.77110014	None	0.414188705	7528
DLBC	YY1	TSCmTOR	0.77110014	None	0.142171227	7528

ESCA	CD274	Apoptosis	0.332102536	None	0.293000203	29126
ESCA	CD274	CellCycle	0.828926996	None	-0.048245264	29126
ESCA	CD274	DNADamage	0.093382513	None	-0.657375131	29126
ESCA	CD274	EMT	0.048008379	Activation	1.243498518	29126
ESCA	CD274	Hormone AR	0.048008379	Inhibition	-0.700533423	29126
ESCA	CD274	Hormone ER	0.22109253	None	0.190848077	29126
ESCA	CD274	PI3KAKT	0.828926996	None	0.06811258	29126
ESCA	CD274	RASMAPK	0.733623304	None	-0.163942824	29126
ESCA	CD274	RTK	0.086193039	None	-0.832049446	29126
ESCA	CD274	TSCmTOR	0.758924838	None	-0.121155583	29126
ESCA	YY1	Apoptosis	0.644208444	None	0.1280769	7528
ESCA	YY1	CellCycle	0.388013077	None	0.268460619	7528
ESCA	YY1	DNADamage	0.450939426	None	0.319758381	7528
ESCA	YY1	EMT	0.450939426	None	0.449643965	7528
ESCA	YY1	Hormone AR	0.134050052	None	-0.517354657	7528
ESCA	YY1	Hormone ER	0.297431149	None	-0.175792964	7528
ESCA	YY1	PI3KAKT	0.909804053	None	-0.031574326	7528
ESCA	YY1	RASMAPK	0.028824908	Inhibition	-0.649918256	7528
ESCA	YY1	RTK	0.028824908	Inhibition	-1.028883304	7528
ESCA	YY1	TSCmTOR	0.028824908	Inhibition	-0.616844405	7528
GBM	CD274	Apoptosis	0.874488672	None	0.117555055	29126
GBM	CD274	CellCycle	0.874488672	None	-0.442440085	29126
GBM	CD274	DNADamage	0.874488672	None	-0.363168809	29126
GBM	CD274	EMT	0.874488672	None	0.174423837	29126

GBM	CD274	Hormone AR	0.874488672	None	-0.163688423	29126
GBM	CD274	Hormone ER	0.874488672	None	-0.053737027	29126
GBM	CD274	PI3KAKT	0.874488672	None	-0.2924957	29126
GBM	CD274	RASMAPK	0.874488672	None	0.180014613	29126
GBM	CD274	RTK	0.874488672	None	0.240229947	29126
GBM	CD274	TSCmTOR	0.961335356	None	0.022384523	29126
GBM	YY1	Apoptosis	0.496483111	None	0.437929037	7528
GBM	YY1	CellCycle	0.688270976	None	0.199104239	7528
GBM	YY1	DNADamage	0.776858913	None	0.074119415	7528
GBM	YY1	EMT	0.688270976	None	0.143452667	7528
GBM	YY1	Hormone AR	0.496483111	None	0.246377535	7528
GBM	YY1	Hormone ER	0.632687086	None	-0.208419159	7528
GBM	YY1	PI3KAKT	0.600490196	None	-0.64300876	7528
GBM	YY1	RASMAPK	0.688270976	None	-0.252834027	7528
GBM	YY1	RTK	0.648348083	None	0.398491261	7528
GBM	YY1	TSCmTOR	0.496483111	None	-0.742758655	7528
HNSC	CD274	Apoptosis	3.45E-07	Activation	1.053554	29126
HNSC	CD274	CellCycle	0.509333154	None	-0.133835792	29126
HNSC	CD274	DNADamage	0.026571424	Inhibition	-0.453819206	29126
HNSC	CD274	EMT	0.002649399	Activation	1.061031904	29126
HNSC	CD274	Hormone AR	0.509918218	None	-0.115938585	29126
HNSC	CD274	Hormone ER	0.685055169	None	0.029235609	29126
HNSC	CD274	PI3KAKT	0.046324861	Inhibition	-0.675739919	29126
HNSC	CD274	RASMAPK	0.191262384	None	-0.275659083	29126

HNSC	CD274	RTK	0.175718195	None	-0.427080087	29126
HNSC	CD274	TSCmTOR	0.191504701	None	-0.283901268	29126
HNSC	YY1	Apoptosis	0.993820643	None	0.007852291	7528
HNSC	YY1	CellCycle	0.449645633	None	0.240311957	7528
HNSC	YY1	DNADamage	0.829956382	None	0.094933611	7528
HNSC	YY1	EMT	0.306942676	None	-0.663258406	7528
HNSC	YY1	Hormone AR	0.993820643	None	-0.001219569	7528
HNSC	YY1	Hormone ER	0.691966537	None	-0.063537466	7528
HNSC	YY1	PI3KAKT	0.676625436	None	-0.316829098	7528
HNSC	YY1	RASMAPK	0.449645633	None	-0.260653007	7528
HNSC	YY1	RTK	0.901442353	None	0.089130609	7528
HNSC	YY1	TSCmTOR	0.691966537	None	0.15493966	7528
KICH	CD274	Apoptosis	0.823091752	None	0.044116862	29126
KICH	CD274	CellCycle	0.612650683	None	0.267342027	29126
KICH	CD274	DNADamage	0.683374034	None	0.296149231	29126
KICH	CD274	EMT	0.472193235	None	0.767634588	29126
KICH	CD274	Hormone AR	0.612650683	None	0.198704858	29126
KICH	CD274	Hormone ER	0.612650683	None	-0.301394671	29126
KICH	CD274	PI3KAKT	0.683374034	None	-0.1683954	29126
KICH	CD274	RASMAPK	0.612650683	None	0.407871046	29126
KICH	CD274	RTK	0.683374034	None	0.189961777	29126
KICH	CD274	TSCmTOR	0.683374034	None	0.142515865	29126
KICH	YY1	Apoptosis	0.63340621	None	-0.251394068	7528
KICH	YY1	CellCycle	0.63340621	None	0.257405547	7528

KICH	YY1	DNADamage	0.63340621	None	0.448072831	7528
KICH	YY1	EMT	0.803415075	None	0.150917251	7528
KICH	YY1	Hormone AR	0.63340621	None	0.169058771	7528
KICH	YY1	Hormone ER	0.746226552	None	-0.144063985	7528
KICH	YY1	PI3KAKT	0.803415075	None	0.098873625	7528
KICH	YY1	RASMAPK	0.63340621	None	0.676611628	7528
KICH	YY1	RTK	0.746226552	None	0.222937485	7528
KICH	YY1	TSCmTOR	0.849986895	None	0.053132683	7528
KIRC	CD274	Apoptosis	0.493916883	None	0.087205137	29126
KIRC	CD274	CellCycle	0.477809766	None	0.126184937	29126
KIRC	CD274	DNADamage	0.38398591	None	0.127287269	29126
KIRC	CD274	EMT	0.979091794	None	0.016909381	29126
KIRC	CD274	Hormone AR	0.27630088	None	0.136040416	29126
KIRC	CD274	Hormone ER	0.38398591	None	0.148680819	29126
KIRC	CD274	PI3KAKT	0.493916883	None	0.153788284	29126
KIRC	CD274	RASMAPK	0.979091794	None	0.005155258	29126
KIRC	CD274	RTK	0.037074043	Activation	0.508057486	29126
KIRC	CD274	TSCmTOR	0.046586945	Inhibition	-0.37626823	29126
KIRC	YY1	Apoptosis	0.904717453	None	-0.037533697	7528
KIRC	YY1	CellCycle	0.853859975	None	-0.077689127	7528
KIRC	YY1	DNADamage	0.642455152	None	-0.115621075	7528
KIRC	YY1	EMT	0.642455152	None	0.179968202	7528
KIRC	YY1	Hormone AR	0.642455152	None	0.089011382	7528
KIRC	YY1	Hormone ER	0.996199208	None	-0.00052041	7528

KIRC	YY1	PI3KAKT	0.904717453	None	-0.040287378	7528
KIRC	YY1	RASMAPK	0.746775829	None	0.175033262	7528
KIRC	YY1	RTK	0.105936419	None	0.44771921	7528
KIRC	YY1	TSCmTOR	0.904717453	None	0.051039093	7528
KIRP	CD274	Apoptosis	0.990759266	None	0.002153211	29126
KIRP	CD274	CellCycle	0.000010871	Activation	0.893965915	29126
KIRP	CD274	DNADamage	0.1722062	None	0.265578526	29126
KIRP	CD274	EMT	0.857609278	None	0.078295848	29126
KIRP	CD274	Hormone AR	0.130375964	None	0.356025669	29126
KIRP	CD274	Hormone ER	2.25E-08	Activation	0.796935251	29126
KIRP	CD274	PI3KAKT	0.043189749	Inhibition	-0.885033627	29126
KIRP	CD274	RASMAPK	0.007323331	Activation	0.540415279	29126
KIRP	CD274	RTK	0.011531397	Activation	0.609768623	29126
KIRP	CD274	TSCmTOR	0.418329112	None	0.242843674	29126
KIRP	YY1	Apoptosis	0.351892934	None	-0.232400455	7528
KIRP	YY1	CellCycle	0.02903522	Activation	0.535281953	7528
KIRP	YY1	DNADamage	0.576808964	None	0.125410221	7528
KIRP	YY1	EMT	0.107396529	None	-0.546072115	7528
KIRP	YY1	Hormone AR	0.003509063	Activation	0.715475648	7528
KIRP	YY1	Hormone ER	0.576808964	None	-0.103552006	7528
KIRP	YY1	PI3KAKT	0.946661107	None	-0.026118582	7528
KIRP	YY1	RASMAPK	0.815289708	None	-0.061434933	7528
KIRP	YY1	RTK	0.030456447	Activation	0.566952992	7528
KIRP	YY1	TSCmTOR	0.351098404	None	-0.338062169	7528

LGG	CD274	Apoptosis	0.590294969	None	0.05934921	29126
LGG	CD274	CellCycle	0.590294969	None	0.089087173	29126
LGG	CD274	DNADamage	0.000020244	Inhibition	-0.643786857	29126
LGG	CD274	EMT	0.246729341	None	0.168307279	29126
LGG	CD274	Hormone AR	0.540044239	None	-0.083038963	29126
LGG	CD274	Hormone ER	0.246738973	None	0.119426242	29126
LGG	CD274	PI3KAKT	0.6505135	None	-0.090204107	29126
LGG	CD274	RASMAPK	0.6505135	None	0.069856322	29126
LGG	CD274	RTK	0.149831634	None	0.390306348	29126
LGG	CD274	TSCmTOR	0.591652555	None	-0.10138575	29126
LGG	YY1	Apoptosis	0.135197868	None	0.1430914	7528
LGG	YY1	CellCycle	0.7003558	None	0.052347316	7528
LGG	YY1	DNADamage	0.000011504	Activation	0.656021371	7528
LGG	YY1	EMT	0.206996751	None	0.145836392	7528
LGG	YY1	Hormone AR	0.135197868	None	0.145536906	7528
LGG	YY1	Hormone ER	0.897229094	None	-0.009378538	7528
LGG	YY1	PI3KAKT	0.228337398	None	0.270279352	7528
LGG	YY1	RASMAPK	0.135197868	None	0.332104611	7528
LGG	YY1	RTK	0.633540073	None	0.121190673	7528
LGG	YY1	TSCmTOR	0.176239535	None	-0.240738748	7528
LIHC	CD274	Apoptosis	0.652348303	None	0.089519902	29126
LIHC	CD274	CellCycle	0.929464272	None	0.015275171	29126
LIHC	CD274	DNADamage	0.652348303	None	0.104350968	29126
LIHC	CD274	EMT	0.281788659	None	0.3173931	29126

LIHC	CD274	Hormone AR	0.581056063	None	0.144838274	29126
LIHC	CD274	Hormone ER	0.281788659	None	0.259988003	29126
LIHC	CD274	PI3KAKT	0.652348303	None	0.127328578	29126
LIHC	CD274	RASMAPK	0.652348303	None	0.155569639	29126
LIHC	CD274	RTK	0.929464272	None	-0.047730253	29126
LIHC	CD274	TSCmTOR	0.929464272	None	0.01478122	29126
LIHC	YY1	Apoptosis	0.897184737	None	-0.032602318	7528
LIHC	YY1	CellCycle	0.865737936	None	0.052306276	7528
LIHC	YY1	DNADamage	0.865737936	None	-0.084229134	7528
LIHC	YY1	EMT	0.897184737	None	-0.021626645	7528
LIHC	YY1	Hormone AR	0.865737936	None	-0.080690897	7528
LIHC	YY1	Hormone ER	0.865737936	None	0.078291109	7528
LIHC	YY1	PI3KAKT	0.302223311	None	0.367494725	7528
LIHC	YY1	RASMAPK	0.897184737	None	-0.033584102	7528
LIHC	YY1	RTK	0.865737936	None	0.118967374	7528
LIHC	YY1	TSCmTOR	0.865737936	None	0.09031499	7528
LUAD	CD274	Apoptosis	0.122811959	None	0.348828814	29126
LUAD	CD274	CellCycle	0.721568319	None	-0.110308683	29126
LUAD	CD274	DNADamage	0.318306825	None	-0.205319733	29126
LUAD	CD274	EMT	2.29E-06	Activation	1.123435793	29126
LUAD	CD274	Hormone AR	0.984874626	None	0.003008624	29126
LUAD	CD274	Hormone ER	0.318306825	None	0.110422869	29126
LUAD	CD274	PI3KAKT	0.750674363	None	-0.096290501	29126
LUAD	CD274	RASMAPK	0.266299253	None	0.345178901	29126

LUAD	CD274	RTK	0.39660684	None	0.254689032	29126
LUAD	CD274	TSCmTOR	0.750674363	None	0.085429216	29126
LUAD	YY1	Apoptosis	0.003552665	Activation	0.521948424	7528
LUAD	YY1	CellCycle	0.000118241	Activation	0.711306148	7528
LUAD	YY1	DNADamage	0.339079828	None	0.169586679	7528
LUAD	YY1	EMT	0.143553645	None	0.416670983	7528
LUAD	YY1	Hormone AR	0.981485943	None	-0.003694469	7528
LUAD	YY1	Hormone ER	0.228736842	None	-0.123870612	7528
LUAD	YY1	PI3KAKT	0.539301236	None	-0.15947603	7528
LUAD	YY1	RASMAPK	0.02465619	Inhibition	-0.524754174	7528
LUAD	YY1	RTK	0.539301236	None	-0.162885141	7528
LUAD	YY1	TSCmTOR	0.339079828	None	-0.209642962	7528
LUSC	CD274	Apoptosis	0.625349939	None	-0.154128799	29126
LUSC	CD274	CellCycle	0.85130031	None	0.028537946	29126
LUSC	CD274	DNADamage	0.85130031	None	-0.028006475	29126
LUSC	CD274	EMT	0.625349939	None	0.199690401	29126
LUSC	CD274	Hormone AR	0.625349939	None	0.122737595	29126
LUSC	CD274	Hormone ER	0.365641576	None	0.104397633	29126
LUSC	CD274	PI3KAKT	0.85130031	None	0.107948751	29126
LUSC	CD274	RASMAPK	0.365641576	None	-0.301004425	29126
LUSC	CD274	RTK	0.85130031	None	-0.048487999	29126
LUSC	CD274	TSCmTOR	0.091358745	None	-0.513621577	29126
LUSC	YY1	Apoptosis	0.048837992	Activation	0.374239029	7528
LUSC	YY1	CellCycle	0.142002565	None	0.288165161	7528

LUSC	YY1	DNADamage	0.309799104	None	-0.191259369	7528
LUSC	YY1	EMT	0.192101892	None	-0.357983742	7528
LUSC	YY1	Hormone AR	0.040182477	Inhibition	-0.36402991	7528
LUSC	YY1	Hormone ER	0.010757901	Inhibition	-0.212757783	7528
LUSC	YY1	PI3KAKT	0.995022983	None	0.0021782	7528
LUSC	YY1	RASMAPK	0.736676175	None	-0.077767567	7528
LUSC	YY1	RTK	0.736676175	None	-0.088107717	7528
LUSC	YY1	TSCmTOR	0.472060533	None	0.191252657	7528
MESO	CD274	Apoptosis	0.823234175	None	0.086430339	29126
MESO	CD274	CellCycle	0.517061857	None	-0.348179284	29126
MESO	CD274	DNADamage	0.336828103	None	-0.620257016	29126
MESO	CD274	EMT	0.356884446	None	-0.560656026	29126
MESO	CD274	Hormone AR	0.580712026	None	-0.214190673	29126
MESO	CD274	Hormone ER	0.823234175	None	-0.069704835	29126
MESO	CD274	PI3KAKT	0.873495255	None	-0.072003295	29126
MESO	CD274	RASMAPK	0.336828103	None	-0.913536418	29126
MESO	CD274	RTK	0.336828103	None	-0.682045204	29126
MESO	CD274	TSCmTOR	0.336828103	None	-0.794934024	29126
MESO	YY1	Apoptosis	0.557198979	None	0.293966184	7528
MESO	YY1	CellCycle	0.972681448	None	0.011940572	7528
MESO	YY1	DNADamage	0.769492355	None	0.238661762	7528
MESO	YY1	EMT	0.064415882	None	-1.098757761	7528
MESO	YY1	Hormone AR	0.769492355	None	-0.101938296	7528
MESO	YY1	Hormone ER	0.434723934	None	-0.349631597	7528

MESO	YY1	PI3KAKT	0.636402462	None	0.450781082	7528
MESO	YY1	RASMAPK	0.557198979	None	-0.56272589	7528
MESO	YY1	RTK	0.769492355	None	0.243543468	7528
MESO	YY1	TSCmTOR	0.769492355	None	-0.206576034	7528
OV	CD274	Apoptosis	0.004195136	Activation	0.611936752	29126
OV	CD274	CellCycle	0.881088593	None	-0.043623314	29126
OV	CD274	DNADamage	0.000315751	Inhibition	-1.023841665	29126
OV	CD274	EMT	0.38961628	None	0.334651894	29126
OV	CD274	Hormone AR	0.38961628	None	-0.228438532	29126
OV	CD274	Hormone ER	0.38961628	None	-0.303950921	29126
OV	CD274	PI3KAKT	0.24124132	None	-0.62872723	29126
OV	CD274	RASMAPK	0.235316329	None	0.663359212	29126
OV	CD274	RTK	0.719467628	None	-0.087333706	29126
OV	CD274	TSCmTOR	0.881088593	None	-0.03237971	29126
OV	YY1	Apoptosis	0.974430777	None	0.29665989	7528
OV	YY1	CellCycle	0.974430777	None	0.101396048	7528
OV	YY1	DNADamage	0.974430777	None	0.110220124	7528
OV	YY1	EMT	0.974430777	None	-0.077654348	7528
OV	YY1	Hormone AR	0.974430777	None	0.077776243	7528
OV	YY1	Hormone ER	0.974430777	None	0.010537123	7528
OV	YY1	PI3KAKT	0.974430777	None	0.052936156	7528
OV	YY1	RASMAPK	0.974430777	None	-0.011458044	7528
OV	YY1	RTK	0.974430777	None	-0.083449676	7528
OV	YY1	TSCmTOR	0.974430777	None	-0.109099867	7528

PAAD	CD274	Apoptosis	0.881638398	None	0.085587719	29126
PAAD	CD274	CellCycle	0.819574068	None	0.242847238	29126
PAAD	CD274	DNADamage	0.881638398	None	0.056560908	29126
PAAD	CD274	EMT	0.819574068	None	0.527774177	29126
PAAD	CD274	Hormone AR	0.819574068	None	0.373745027	29126
PAAD	CD274	Hormone ER	0.881638398	None	0.058013862	29126
PAAD	CD274	PI3KAKT	0.881638398	None	-0.072981172	29126
PAAD	CD274	RASMAPK	0.881638398	None	0.154140543	29126
PAAD	CD274	RTK	0.669243256	None	0.477836687	29126
PAAD	CD274	TSCmTOR	0.881638398	None	0.082226868	29126
PAAD	YY1	Apoptosis	0.10443571	None	0.700427831	7528
PAAD	YY1	CellCycle	0.023560321	Activation	0.633446784	7528
PAAD	YY1	DNADamage	0.814105049	None	0.088260386	7528
PAAD	YY1	EMT	0.727436737	None	0.227258328	7528
PAAD	YY1	Hormone AR	0.727436737	None	0.185273053	7528
PAAD	YY1	Hormone ER	0.727436737	None	0.092167387	7528
PAAD	YY1	PI3KAKT	0.22914465	None	-0.653120916	7528
PAAD	YY1	RASMAPK	0.727436737	None	0.150239555	7528
PAAD	YY1	RTK	0.22914465	None	0.441638649	7528
PAAD	YY1	TSCmTOR	0.614341193	None	-0.293402367	7528
PCPG	CD274	Apoptosis	0.516434605	None	0.382192887	29126
PCPG	CD274	CellCycle	0.898997747	None	-0.069892942	29126
PCPG	CD274	DNADamage	0.086152231	None	-0.726087763	29126
PCPG	CD274	EMT	0.905150798	None	0.023116282	29126

PCPG	CD274	Hormone AR	0.475572122	None	-0.393394225	29126
PCPG	CD274	Hormone ER	0.551969287	None	-0.13873325	29126
PCPG	CD274	PI3KAKT	0.898997747	None	-0.098043812	29126
PCPG	CD274	RASMAPK	0.086152231	None	0.836585965	29126
PCPG	CD274	RTK	0.475572122	None	0.294440726	29126
PCPG	CD274	TSCmTOR	0.234019761	None	0.825815975	29126
PCPG	YY1	Apoptosis	0.870454964	None	-0.057779266	7528
PCPG	YY1	CellCycle	0.2760708	None	0.455084256	7528
PCPG	YY1	DNADamage	0.43953937	None	0.340369436	7528
PCPG	YY1	EMT	0.2760708	None	-0.36532698	7528
PCPG	YY1	Hormone AR	0.43953937	None	0.352599904	7528
PCPG	YY1	Hormone ER	0.783295315	None	0.096939952	7528
PCPG	YY1	PI3KAKT	0.783295315	None	0.216991097	7528
PCPG	YY1	RASMAPK	0.43953937	None	0.416785868	7528
PCPG	YY1	RTK	0.391321162	None	0.388820595	7528
PCPG	YY1	TSCmTOR	0.870454964	None	0.124558405	7528
PRAD	CD274	Apoptosis	0.515527655	None	-0.055279246	29126
PRAD	CD274	CellCycle	0.219637717	None	-0.115193583	29126
PRAD	CD274	DNADamage	0.00151701	Inhibition	-0.429102414	29126
PRAD	CD274	EMT	0.05836006	None	0.424575362	29126
PRAD	CD274	Hormone AR	0.007641323	Inhibition	-0.384091758	29126
PRAD	CD274	Hormone ER	0.338849127	None	0.063886996	29126
PRAD	CD274	PI3KAKT	0.041241521	Activation	0.534344604	29126
PRAD	CD274	RASMAPK	0.007641323	Activation	0.601817823	29126

PRAD	CD274	RTK	0.007641323	Activation	0.420161555	29126
PRAD	CD274	TSCmTOR	0.007641323	Activation	0.542483317	29126
PRAD	YY1	Apoptosis	0.000046959	Activation	0.374064275	7528
PRAD	YY1	CellCycle	0.042710354	Activation	0.175060079	7528
PRAD	YY1	DNADamage	0.000276226	Activation	0.44456719	7528
PRAD	YY1	EMT	0.007817218	Inhibition	-0.586253445	7528
PRAD	YY1	Hormone AR	0.000046959	Activation	0.559550834	7528
PRAD	YY1	Hormone ER	0.612425471	None	-0.03181523	7528
PRAD	YY1	PI3KAKT	0.004445962	Inhibition	-0.723730691	7528
PRAD	YY1	RASMAPK	0.000276226	Inhibition	-0.799040758	7528
PRAD	YY1	RTK	0.009441939	Inhibition	-0.383969516	7528
PRAD	YY1	TSCmTOR	0.030133164	Inhibition	-0.421601499	7528
READ	CD274	Apoptosis	0.907752254	None	-0.050830322	29126
READ	CD274	CellCycle	0.298967208	None	0.485974652	29126
READ	CD274	DNADamage	0.067419837	None	-0.982397533	29126
READ	CD274	EMT	0.247004123	None	1.021648989	29126
READ	CD274	Hormone AR	0.226096886	None	-0.635250107	29126
READ	CD274	Hormone ER	0.247004123	None	0.248821936	29126
READ	CD274	PI3KAKT	0.068563974	None	1.390924084	29126
READ	CD274	RASMAPK	0.214621829	None	1.030336794	29126
READ	CD274	RTK	0.421254004	None	0.290505793	29126
READ	CD274	TSCmTOR	0.247004123	None	0.435821212	29126
READ	YY1	Apoptosis	0.951510804	None	0.724565306	7528
READ	YY1	CellCycle	0.993534132	None	0.04255604	7528

READ	YY1	DNADamage	0.993534132	None	0.109331593	7528
READ	YY1	EMT	0.993534132	None	-0.321062857	7528
READ	YY1	Hormone AR	0.993534132	None	0.119559366	7528
READ	YY1	Hormone ER	0.993534132	None	-0.050205104	7528
READ	YY1	PI3KAKT	0.993534132	None	0.047287453	7528
READ	YY1	RASMAPK	0.993534132	None	-0.20507733	7528
READ	YY1	RTK	0.993534132	None	-0.063281173	7528
READ	YY1	TSCmTOR	0.993534132	None	0.002607055	7528
SARC	CD274	Apoptosis	0.93313871	None	-0.027492193	29126
SARC	CD274	CellCycle	0.148949995	None	0.46977906	29126
SARC	CD274	DNADamage	0.000255203	Inhibition	-1.028888996	29126
SARC	CD274	EMT	0.148949995	None	-0.385045074	29126
SARC	CD274	Hormone AR	0.436833586	None	-0.248134308	29126
SARC	CD274	Hormone ER	0.93313871	None	0.023223765	29126
SARC	CD274	PI3KAKT	0.148949995	None	0.71992665	29126
SARC	CD274	RASMAPK	0.249795467	None	0.369051245	29126
SARC	CD274	RTK	0.877129482	None	0.111476992	29126
SARC	CD274	TSCmTOR	0.93313871	None	-0.018694037	29126
SARC	YY1	Apoptosis	0.71846428	None	0.076660834	7528
SARC	YY1	CellCycle	0.000582709	Activation	0.925763962	7528
SARC	YY1	DNADamage	0.38423092	None	-0.356404142	7528
SARC	YY1	EMT	0.493824684	None	-0.235493543	7528
SARC	YY1	Hormone AR	0.752530289	None	-0.069892968	7528
SARC	YY1	Hormone ER	0.026445907	Inhibition	-0.422038216	7528

SARC	YY1	PI3KAKT	0.077927281	None	0.87064359	7528
SARC	YY1	RASMAPK	0.71846428	None	-0.156337428	7528
SARC	YY1	RTK	0.71846428	None	-0.122147803	7528
SARC	YY1	TSCmTOR	0.71846428	None	0.139626652	7528
SKCM	CD274	Apoptosis	0.933964802	None	0.463314959	29126
SKCM	CD274	CellCycle	0.933964802	None	0.118430424	29126
SKCM	CD274	DNADamage	0.933964802	None	0.249102833	29126
SKCM	CD274	EMT	0.933964802	None	-0.302931928	29126
SKCM	CD274	Hormone AR	0.933964802	None	0.053855554	29126
SKCM	CD274	Hormone ER	0.933964802	None	0.057153748	29126
SKCM	CD274	PI3KAKT	0.933964802	None	0.317085294	29126
SKCM	CD274	RASMAPK	0.933964802	None	0.027320337	29126
SKCM	CD274	RTK	0.933964802	None	-0.020202124	29126
SKCM	CD274	TSCmTOR	0.933964802	None	0.219525907	29126
SKCM	YY1	Apoptosis	0.863125225	None	-0.094865998	7528
SKCM	YY1	CellCycle	0.960528657	None	0.014147123	7528
SKCM	YY1	DNADamage	0.482409068	None	0.340759819	7528
SKCM	YY1	EMT	0.557097149	None	-0.374789329	7528
SKCM	YY1	Hormone AR	0.241969675	None	0.497575921	7528
SKCM	YY1	Hormone ER	0.863125225	None	0.045976968	7528
SKCM	YY1	PI3KAKT	0.863125225	None	0.172576857	7528
SKCM	YY1	RASMAPK	0.241969675	None	-0.670339841	7528
SKCM	YY1	RTK	0.241969675	None	-0.416658613	7528
SKCM	YY1	TSCmTOR	0.685327431	None	-0.277582087	7528

STAD	CD274	Apoptosis	0.000041802	Activation	0.988794566	29126
STAD	CD274	CellCycle	0.23454213	None	0.293436313	29126
STAD	CD274	DNADamage	0.160331743	None	-0.325974856	29126
STAD	CD274	EMT	0.822900041	None	0.061193963	29126
STAD	CD274	Hormone AR	0.068594606	None	-0.473022667	29126
STAD	CD274	Hormone ER	0.160331743	None	0.107515932	29126
STAD	CD274	PI3KAKT	0.759464558	None	-0.094014873	29126
STAD	CD274	RASMAPK	0.759464558	None	-0.09072165	29126
STAD	CD274	RTK	0.156712934	None	-0.423014771	29126
STAD	CD274	TSCmTOR	0.068594606	None	-0.448353446	29126
STAD	YY1	Apoptosis	0.120074384	None	0.410353859	7528
STAD	YY1	CellCycle	0.009156023	Activation	0.65146774	7528
STAD	YY1	DNADamage	0.05452706	None	0.439411907	7528
STAD	YY1	EMT	0.00101054	Inhibition	-1.048710588	7528
STAD	YY1	Hormone AR	0.365786538	None	0.246084147	7528
STAD	YY1	Hormone ER	0.053433073	None	-0.15514613	7528
STAD	YY1	PI3KAKT	0.680347035	None	0.117824049	7528
STAD	YY1	RASMAPK	0.680347035	None	-0.100252258	7528
STAD	YY1	RTK	0.981431083	None	0.005259115	7528
STAD	YY1	TSCmTOR	0.37539521	None	0.216896675	7528
TGCT	CD274	Apoptosis	0.00045231	Activation	0.851549673	29126
TGCT	CD274	CellCycle	0.464544729	None	0.180011055	29126
TGCT	CD274	DNADamage	0.006559953	Activation	0.984852207	29126
TGCT	CD274	EMT	0.464544729	None	0.212312985	29126

TGCT	CD274	Hormone AR	0.042449824	Activation	0.514779966	29126
TGCT	CD274	Hormone ER	0.000013359	Activation	0.82521438	29126
TGCT	CD274	PI3KAKT	0.230375298	None	-0.289974405	29126
TGCT	CD274	RASMAPK	0.015699853	Inhibition	-0.627669824	29126
TGCT	CD274	RTK	0.012545873	Inhibition	-0.493152553	29126
TGCT	CD274	TSCmTOR	0.080566692	None	-0.306838587	29126
TGCT	YY1	Apoptosis	0.004945547	Activation	0.67984029	7528
TGCT	YY1	CellCycle	0.000059832	Activation	1.0658646	7528
TGCT	YY1	DNADamage	0.00827304	Activation	0.914632513	7528
TGCT	YY1	EMT	0.029065479	Inhibition	-0.64557762	7528
TGCT	YY1	Hormone AR	0.101575815	None	0.403091404	7528
TGCT	YY1	Hormone ER	0.003334638	Activation	0.594044367	7528
TGCT	YY1	PI3KAKT	0.457872041	None	0.16240055	7528
TGCT	YY1	RASMAPK	0.004077929	Inhibition	-0.756350369	7528
TGCT	YY1	RTK	0.026215497	Inhibition	-0.42480271	7528
TGCT	YY1	TSCmTOR	0.183088194	None	-0.224546911	7528
THCA	CD274	Apoptosis	0.000886609	Activation	0.401747108	29126
THCA	CD274	CellCycle	0.091161737	None	-0.301296044	29126
THCA	CD274	DNADamage	0.000034167	Inhibition	-0.505901643	29126
THCA	CD274	EMT	0.000031729	Activation	1.104645749	29126
THCA	CD274	Hormone AR	0.000058146	Inhibition	-0.923052944	29126
THCA	CD274	Hormone ER	0.909486854	None	0.011609464	29126
THCA	CD274	PI3KAKT	0.000125825	Inhibition	-0.878446671	29126
THCA	CD274	RASMAPK	0.05035394	None	-0.337143533	29126

THCA	CD274	RTK	0.000243086	Inhibition	-0.564606539	29126
THCA	CD274	TSCmTOR	0.088113289	None	-0.312735204	29126
THCA	YY1	Apoptosis	0.064101458	None	-0.312290687	7528
THCA	YY1	CellCycle	0.609835683	None	0.105198659	7528
THCA	YY1	DNADamage	0.371110427	None	-0.182099536	7528
THCA	YY1	EMT	0.476557113	None	-0.252459401	7528
THCA	YY1	Hormone AR	0.609835683	None	0.140681948	7528
THCA	YY1	Hormone ER	0.414075331	None	-0.14414755	7528
THCA	YY1	PI3KAKT	0.476557113	None	-0.215486602	7528
THCA	YY1	RASMAPK	0.888018454	None	0.022716518	7528
THCA	YY1	RTK	0.371110427	None	-0.26328452	7528
THCA	YY1	TSCmTOR	0.434284528	None	-0.21550691	7528
THYM	CD274	Apoptosis	0.126220305	None	0.359278003	29126
THYM	CD274	CellCycle	0.074885822	None	-0.640566226	29126
THYM	CD274	DNADamage	0.074885822	None	-0.486670053	29126
THYM	CD274	EMT	0.55613437	None	0.168314295	29126
THYM	CD274	Hormone AR	0.013645178	Inhibition	-0.83000138	29126
THYM	CD274	Hormone ER	0.366532955	None	0.136410321	29126
THYM	CD274	PI3KAKT	0.074885822	None	0.638807854	29126
THYM	CD274	RASMAPK	0.00895465	Activation	0.92260152	29126
THYM	CD274	RTK	0.074885822	None	0.412196497	29126
THYM	CD274	TSCmTOR	0.126220305	None	0.30179853	29126
THYM	YY1	Apoptosis	0.834946293	None	-0.242628797	7528
THYM	YY1	CellCycle	0.480254952	None	0.605514798	7528

THYM	YY1	DNADamage	0.960216737	None	0.034682383	7528
THYM	YY1	EMT	0.93129656	None	-0.180456797	7528
THYM	YY1	Hormone AR	0.834946293	None	0.272508134	7528
THYM	YY1	Hormone ER	0.699459771	None	-0.204800393	7528
THYM	YY1	PI3KAKT	0.93129656	None	-0.182010871	7528
THYM	YY1	RASMAPK	0.960216737	None	-0.065314373	7528
THYM	YY1	RTK	0.960216737	None	-0.013532786	7528
THYM	YY1	TSCmTOR	0.960216737	None	0.009239218	7528
UCEC	CD274	Apoptosis	0.001037857	Activation	1.09343849	29126
UCEC	CD274	CellCycle	0.968334642	None	-0.148527656	29126
UCEC	CD274	DNADamage	0.171029479	None	-0.53250477	29126
UCEC	CD274	EMT	0.439747356	None	0.496899804	29126
UCEC	CD274	Hormone AR	0.995659738	None	-0.001105325	29126
UCEC	CD274	Hormone ER	0.769354337	None	-0.260341028	29126
UCEC	CD274	PI3KAKT	0.171029479	None	-1.109264939	29126
UCEC	CD274	RASMAPK	0.551060643	None	-0.439240678	29126
UCEC	CD274	RTK	0.995659738	None	-0.007553196	29126
UCEC	CD274	TSCmTOR	0.992874757	None	-0.066011927	29126
UCEC	YY1	Apoptosis	0.459987382	None	-0.277308522	7528
UCEC	YY1	CellCycle	0.030219927	Activation	0.869271968	7528
UCEC	YY1	DNADamage	0.020554791	Activation	0.785129611	7528
UCEC	YY1	EMT	0.459987382	None	0.390696438	7528
UCEC	YY1	Hormone AR	0.683272228	None	0.10252241	7528
UCEC	YY1	Hormone ER	0.030219927	Inhibition	-0.887428458	7528

UCEC	YY1	PI3KAKT	0.020554791	Inhibition	-1.53957862	7528
UCEC	YY1	RASMAPK	0.418108314	None	-0.504362117	7528
UCEC	YY1	RTK	0.470169613	None	-0.255424261	7528
UCEC	YY1	TSCmTOR	0.947852235	None	-0.016619956	7528
UCS	CD274	Apoptosis	0.478288092	None	0.331939639	29126
UCS	CD274	CellCycle	0.860763015	None	-0.138438598	29126
UCS	CD274	DNADamage	0.175585591	None	-1.29638	29126
UCS	CD274	EMT	0.175585591	None	-1.158720101	29126
UCS	CD274	Hormone AR	0.238546627	None	-0.976277116	29126
UCS	CD274	Hormone ER	0.382469559	None	0.322889965	29126
UCS	CD274	PI3KAKT	0.890293765	None	-0.100839315	29126
UCS	CD274	RASMAPK	0.017965371	Activation	1.204097876	29126
UCS	CD274	RTK	0.238546627	None	0.643935634	29126
UCS	CD274	TSCmTOR	0.890293765	None	0.08824649	29126
UCS	YY1	Apoptosis	0.772812023	None	-0.249450192	7528
UCS	YY1	CellCycle	0.992049754	None	0.008412456	7528
UCS	YY1	DNADamage	0.992049754	None	-0.006940108	7528
UCS	YY1	EMT	0.657421599	None	-0.678880447	7528
UCS	YY1	Hormone AR	0.808161206	None	-0.278256575	7528
UCS	YY1	Hormone ER	0.772812023	None	-0.167736856	7528
UCS	YY1	PI3KAKT	0.657421599	None	0.86858377	7528
UCS	YY1	RASMAPK	0.772812023	None	0.283041801	7528
UCS	YY1	RTK	0.546534547	None	0.665470952	7528
UCS	YY1	TSCmTOR	0.462559227	None	1.013862329	7528

UVM	CD274	Apoptosis	0.472851639	None	0.85536895	29126
UVM	CD274	CellCycle	0.962753496	None	-0.028528668	29126
UVM	CD274	DNADamage	0.310251498	None	1.407516799	29126
UVM	CD274	EMT	0.887986361	None	-0.168524292	29126
UVM	CD274	Hormone AR	0.125557028	None	1.176704631	29126
UVM	CD274	Hormone ER	0.653300685	None	0.445060951	29126
UVM	CD274	PI3KAKT	0.920984206	None	-0.26779874	29126
UVM	CD274	RASMAPK	0.653300685	None	-0.578863311	29126
UVM	CD274	RTK	0.706026488	None	-0.419817529	29126
UVM	CD274	TSCmTOR	0.887986361	None	0.475628807	29126
UVM	YY1	Apoptosis	0.586596555	None	0.514825123	7528
UVM	YY1	CellCycle	0.318478595	None	0.885022816	7528
UVM	YY1	DNADamage	0.586596555	None	0.880152557	7528
UVM	YY1	EMT	0.741494226	None	-0.24781119	7528
UVM	YY1	Hormone AR	0.318478595	None	1.071910794	7528
UVM	YY1	Hormone ER	0.586596555	None	0.312036528	7528
UVM	YY1	PI3KAKT	0.586596555	None	-0.810125241	7528
UVM	YY1	RASMAPK	0.318478595	None	-0.823783346	7528
UVM	YY1	RTK	0.741494226	None	-0.142999588	7528
UVM	YY1	TSCmTOR	0.741494226	None	-0.551877903	7528

ACC: Adrenocortical carcinoma; BLCA: Bladder Urothelial Carcinoma; BRCA: Breast invasive carcinoma; CESC: Cervical squamous cell carcinoma and endocervical adenocarcinoma; CHOL: Cholangio carcinoma; COAD: Colon adenocarcinoma; DLBC: Lymphoid Neoplasm Diffuse Large B-cell Lymphoma; ESCA: Esophageal carcinoma; FDR: false discovery rate; GBM: Glioblastoma multiforme; HNSC: Head and Neck squamous cell carcinoma; KICH: Kidney Chromophobe; KIRC: Kidney renal clear cell carcinoma; KIRP: Kidney renal papillary cell carcinoma; LGG: Brain Lower Grade Glioma; LIHC: Liver hepatocellular carcinoma; LUAD: Lung adenocarcinoma; LUSC: Lung squamous cell carcinoma; MESO: Mesothelioma; OV: Ovarian serous cystadenocarcinoma; PAAD Pancreatic adenocarcinoma; PCPG: Pheochromocytoma and Paraganglioma; PRAD: Prostate adenocarcinoma; READ: Rectum adenocarcinoma; SARC: Sarcoma; SKCM: Skin Cutaneous Melanoma; STAD: Stomach adenocarcinoma; TGCT: Testicular Germ Cell

Tumors; THCA: Thyroid carcinoma; THYM: Thymoma; UCEC: Uterine Corpus Endometrial Carcinoma; UCS: Uterine Carcinosarcoma; UVM: Uveal Melanoma.
